# Rational dsRNA design, scalable production and nanodelivery to enhance spray-induced gene silencing

**DOI:** 10.1038/s42003-026-10663-5

**Published:** 2026-07-14

**Authors:** Guoning Zhu, Chao Xu, Tao Zhang

**Affiliations:** 1 State Key Laboratory of Ecological Safety and Sustainable Development in Arid Lands, Xinjiang Institute of Ecology and Geography, Chinese Academy of Sciences, Urumqi, China; 2 Institute of Germplasm Resources and Biotechnology, Tianjin Academy of Agricultural Sciences, Tianjin, China; 3 State Key Laboratory of Vegetable Biobreeding, Tianjin Academy of Agricultural Sciences, Tianjin, China

**Keywords:** siRNAs, Molecular engineering in plants

## Abstract

Spray-induced gene silencing (SIGS) provides a sustainable, highly targeted alternative to chemical pesticides. This review summarizes recent advances in dsRNA technology through a design-to-delivery framework. Key focuses include bioinformatics-driven multi-target dsRNA design for improved stability and efficacy, cost-effective microbial and cell-free synthesis platforms for scalable production, and nanocarriers that protect dsRNA from degradation while enhancing delivery. Integrating rational design, efficient production, and precision nanodelivery will accelerate SIGS from laboratory concept to practical, eco-friendly tool for global crop protection and sustainable agriculture.

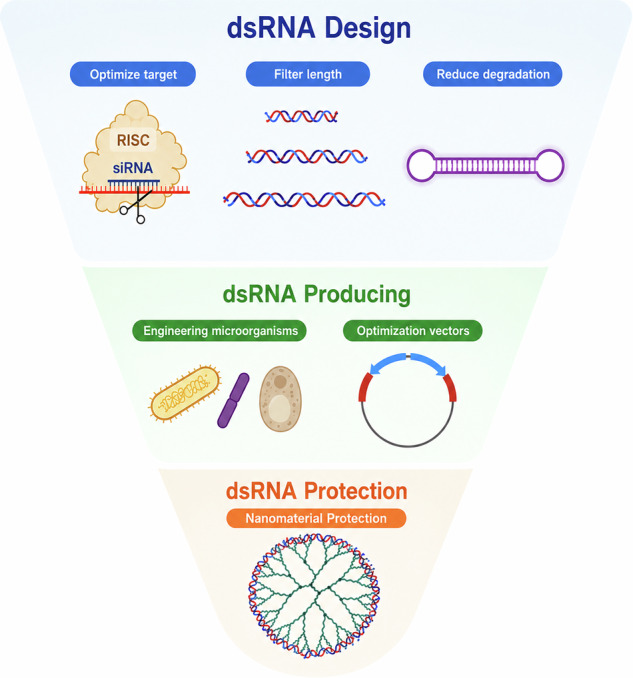

## Abstract (web summary)

A review summarizes advances in spray-induced gene silencing (SIGS), covering rational dsRNA design for efficacy, scalable microbial/cell-free production, and nanodelivery systems for stable, effective crop protection.

## Introduction

Agricultural productivity faces severe threats from a spectrum of pathogens and pests, resulting in substantial reductions in global crop yields^1^ and cumulative annual economic losses estimated at up to $220 billion^2^. To defend against these constant biotic invasions, modern agriculture heavily relies on the extensive application of chemical pesticides. However, the intensive and widespread use of these compounds poses significant potential risks to human health, beneficial non-target organisms, and the environment^3,4^. Therefore, highly efficient, nonpolluting, highly targeted, and human-harmless methods for pest and pathogen control are urgently needed.

Double-stranded RNA (dsRNA)-induced gene silencing is a conserved mechanism fundamental to the defense systems of plants, animals, and fungi. The initial exploration of this mechanism began with the biological function of antisense RNA, which laid the groundwork for the eventual discovery of RNAi. Early studies demonstrated that introducing antisense RNA transcripts (RNA strands complementary to the mRNA) into cells could lead to a reduction in the expression of corresponding genes. For example, in 1984, the injection of artificially synthesized antisense RNA targeting the herpes simplex virus thymidine kinase (TK) gene into mouse L cells (a fibroblast line) resulted in the inhibition of TK gene expression. This phenomenon was initially attributed to the complementary antisense and sense strands physically blocking mRNA translation^5^. These findings provide further insight into RNA-mediated regulation. Notably, in 1990, the unexpected phenomenon of cosuppression was observed in petunias, where the overexpression of the chalcone synthase (CHS) gene, rather than increasing flower color, surprisingly blocked the anthocyanin synthesis pathway, resulting in white or light-colored flowers^6^. Similarly, in 1992, the suppression of an endogenous homologous gene following the introduction of an exogenous homologous gene was noted in fungi, a process termed quelling^7^. The discovery of the first microRNA (miRNA) in 1993, lin-4, a noncoding small RNA in *C. elegans*, was found to reduce lin-14 gene expression through RNA complementarity, further confirming the idea that small nucleic acids play a critical regulatory role^8^. These early observations collectively hint at the critical, yet undefined, role of double-stranded nucleic acids in gene silencing. The understanding of the core mechanism shifted significantly after 1995, when a study demonstrated that injecting either sense or antisense RNA into *C. elegans* could cause the silencing of the par-1 gene^9^. This perplexing finding led scientists to re-examine the mechanism involved. It was not until groundbreaking discovery in 1998 that the gene silencing efficiency of dsRNA was two orders of magnitude greater than that of single-stranded RNA. This established that the previously observed single-strand-mediated silencing was due to trace contamination by unpurified dsRNA, leading to the formal proposal of the concept of RNAi^10^. Since this landmark study, RNAi has been continuously validated across diverse studies, and the underlying pathways in plants, animals, and fungi have been elucidated. The significance of this discovery was recognized with the 2006 Nobel Prize in Physiology or Medicine awarded to Andrew Fire and Craig Mello for the discovery of RNAi. Furthermore, the foundational work on small regulatory RNAs was honored when Victor Ambros was awarded the 2024 Nobel Prize in Physiology or Medicine for the discovery of miRNAs.

## Application of RNAi technology in crop pests and disease control

The core of RNAi technology lies in its mechanism of sequence-specific gene silencing. This conserved cellular process begins with the intracellular processing of dsRNA into a series of short, defined-length small RNA fragments, such as small interfering RNAs (siRNAs) or miRNAs. These fragments subsequently associate with protein complexes, notably Dicer and Argonaute, to assemble the RNA-induced silencing complex (RISC)^11^. The activated RISC complex then functions to recognize and specifically cleave the complementary target mRNA, thereby preventing its translation into protein and ultimately achieving effective gene silencing.

**Fig. 1:**
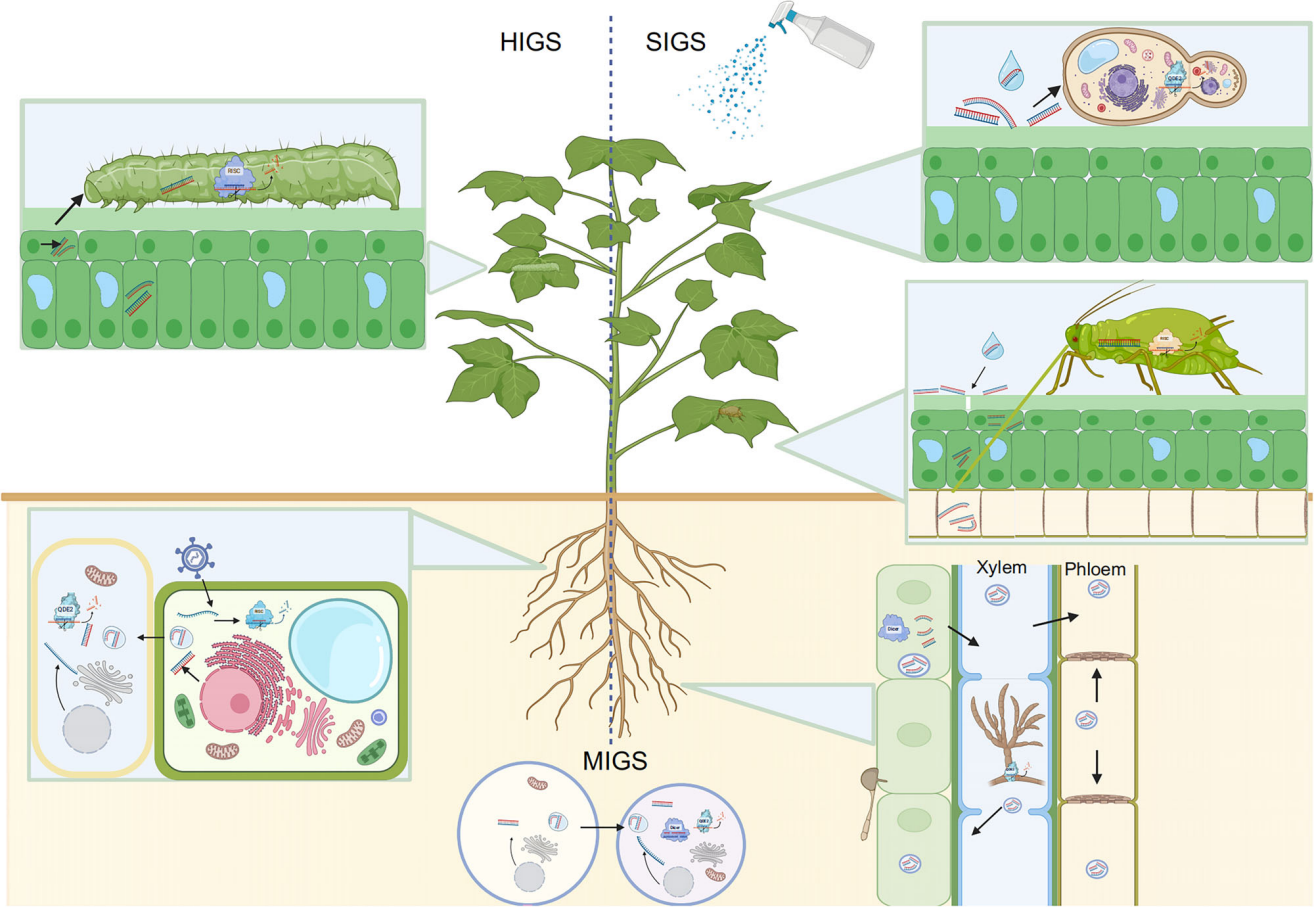
RNAi mechanisms of HIGS, SIGS and MIGS. **HIGS**: dsRNA targeting key genes of pests or pathogens is expressed endogenously in plants. Upon ingestion by herbivorous insects or infection by pathogens, the dsRNA is processed into siRNAs by the Dicer/RISC complex, thereby triggering an RNAi effect against the target organisms. **SIGS**: Exogenous dsRNA or siRNA targeting pests or pathogens is applied via foliar spray. These molecules can be taken up by plant cells and subsequently acquired by pests and pathogens, or they can be taken up directly, thereby triggering an RNAi effect against the target organisms. **MIGS**: Rhizosphere or endophytic microorganisms serve as bio-factories to continuously produce and release dsRNA targeting pests or pathogens. These dsRNAs are ultimately delivered to the pests or to pathogens infecting the plants, thereby triggering an RNAi effect against the target organisms.

In the field of plant pest and disease control, four main implementation methods have evolved into distinct RNAi application paradigms: virus-induced gene silencing (VIGS), host-induced gene silencing (HIGS), spray-induced gene silencing (SIGS), and microbe-induced gene silencing (MIGS) (Fig. 1). Specifically, VIGS utilizes an engineered viral vector carrying a fragment of the target gene to infect the plant; dsRNA is produced during viral replication or transcription within the host cell and subsequently cleaved into various lengths of siRNA, which then exerts a silencing effect^12,13^. Furthermore, this technology has been further extended to enable gene expression/overexpression, moving beyond simple gene silencing^14^. SIGS is an exogenous application strategy in which dsRNA or amiRNA, synthesized in vitro or in vivo, is directly sprayed onto crops. A portion of the applied RNA persists on the plant surface, whereas another portion can penetrate plant cells through stomata and be transported throughout various plant parts^15,16^. Although SIGS still faces limitations in terms of RNA stability and cellular uptake efficiency, it eliminates the need for time-consuming transgenic plant breeding, increasing the speed and flexibility of its application. In contrast, HIGS is a transgenic strategy that involves the creation of stable plant lines capable of constitutively synthesizing specific dsRNA or artificial miRNA (amiRNA) sequences^17–19^. These transgenic plants autonomously express dsRNA or amiRNAs that target the pathogen or pest, thus achieving inherent control objectives. MIGS is an emerging biocontrol approach that transforms harmless soil fungi (primarily common biocontrol agents) into engineered strains capable of synthesizing specific dsRNA or amiRNA sequences. These strains retain their original biocontrol function while simultaneously enhancing resistance against specific pathogens via the RNAi pathway^20,21^.

Currently, HIGS and SIGS represent the most widely utilized RNAi technologies in plant pest and disease control research and practical applications. For example, HIGS has been successfully employed to create transgenic lines of apple, cotton, potato, rice, sugarcane, tomato, and cotton that exhibit robust resistance to pathogens^22–30^. Moreover, SIGS has demonstrated significant potential in crop protection by silencing key growth and development genes in numerous pests and pathogens, including *Fusarium oxysporum*, Botrytis cinerea, Bean Golden Mosaic Virus, Magnaporthe oryzae, Sogatella furcifera, pollen beetles, Sitobion avenae, and Phyllotreta striolata^31–38^ (Table 1).

**Table 1:** Advances in research on RNAi technology in disease control and prevention

Pathogen	Host plant	Target gene	Results	Field test	Methods	Reference
*Diplocarpon coronariae*	apple	*Hsp90*	Detached leaf assays showed reduced disease symptoms in transgenic lines compared to wild-type controls.	No	HIGS	^22^
*Phytophthora infestans*	potato	*ALS*	The downregulation of the ALS gene resulted in significantly increased resistance to Late Blight.	Yes	HIGS	^23^
*Aphis gossypii*	cotton	*AgDPPS1*	The efficacy of the treatment increased cotton aphid mortality rates substantially under both laboratory and field conditions.	Yes	HIGS	^24^
*Fusarium fujikuroi*	rice	*FfCna1, FfCnb1*	The plant exhibited increased resistance to rice Bakanae Disease and displayed a mitigation of disease symptoms.	No	HIGS	^25^
*Fusarium sacchari*	Sugarcane	*FsCYP51*	The transgenic sugarcane lines demonstrated robust resistance against Fusarium infection.	Yes	HIGS	^26^
*Sporisorium scitamineum*	Sugarcane	*SsGlcP*	Transgenic sugarcane lines demonstrated a significant reduction in the biomass of the endophytic basidiomycete.	Yes	HIGS	^27^
*Verticillium dahliae*	cotton	*VdThit*	The transgenic RNAi cotton lines exhibited mitigated disease symptoms accompanied by a significant reduction in Verticillium dahliae biomass.	Yes	HIGS	^28^
*Rhizoctonia solani* AG1-IA	rice	*AGLIP1*	The treatment resulted in the suppression of mycelial growth and biomass, leading to a 57.3% reduction in lesion area on the host plant.	No	HIGS	^29^
*Fusarium oxysporum*f. sp.*Lycopersici*	tomato	*FoFLP1*, *FoFLP4*, *FoFLP5*	A decrease in the Disease Index was observed, correlating with a 2.4- to 7.5-fold reduction in FoFLP gene expression within the pathogen.	No	HIGS	^30^
*Fusarium oxysporum* f.sp. *Lycopersici*	tomato	*FolRDR1*	The treatment resulted in the attenuation of mycelial growth and a reduction in biomass for *Fusarium oxysporum*, and markedly suppressed the growth of Botrytis cinerea.	No	SIGS	^31^
*Botrytis cinerea*	Tobacco, tomato	*BcTRE1*	The significant suppression of Botrytis cinerea growth enhanced the plant’s resistance to Gray Mold disease.	No	SIGS	^32^
*Leaf curl disease-causing begomoviruses*	Tobacco	Av1, Av2, Ac1, Ac4	The method led to a significant decrease in viral accumulation within tobacco plants, resulting in the mitigation of the characteristic leaf-curling symptoms.	No	SIGS	^33^
*Magnaporthe oryzae*	rice	*MoDES1*	The treatment resulted in the significant suppression of Magnaporthe oryzae growth and a corresponding decrease in the lesion area on rice plants.	No	SIGS	^34^
*Sogatella furcifera*	rice	*sfAkt*	The treatment resulted in the significant suppression of female ovarian development, which led to a reduction in egg-laying capacity, correlating with a decrease in target gene expression levels.	No	SIGS	^35^
*Brassicogethes aeneus*	Oilseed rape	*αCOP*	The decrease in target gene expression correlated with a 16% mortality rate observed 15 days after initiation of feeding.	No	SIGS	^36^
*Sitobion avanae*	wheat	*CHS1*	Aphid CHS1 gene expression decreased by 45.32%–50.29%; concurrently, both the total number of aphids and the number of molting aphids were significantly reduced, leading to an increase in wheat grain weight.	No	SIGS	^37^
*Phyllotreta striolata*	Chinese cabbage	*psJHBP-like*, *psaaNAT-like*	Reduced gene expression resulted in a decrease in feeding rate	No	SIGS	^38^
*Verticillium dahliae*	cotton	*PMT*	The treatment inhibited the growth of Verticillium dahliae, leading to alleviated symptoms of cotton Verticillium Wilt.	No	MIGS	^20^

Despite the robust efficacy observed in laboratory settings, the transition of HIGS and SIGS from lab to field continues to face notable performance gaps. Although significant gene silencing and phenotypic resistance have been reported in many studies, the reproducibility of these results is often limited by inconsistencies in dsRNA internalization, environmental degradation, and the inherent variability of RNAi machinery across different pathogens. SIGS, in particular, offers the advantage of bypassing transgenic intervention but faces heightened challenges in maintaining dsRNA stability and ensuring effective penetration under field conditions. Such challenges will require optimization to develop SIGS as a robust and reliable platform for sustainable agricultural biosecurity.

## Rational design of dsRNA for SIGS

HIGS technology necessitates the creation of transgenic crop varieties, a process that is often time-consuming for most crop species. Acquiring stable transgenic plants through tissue culture typically requires anywhere from 6 - 12 months, with additional time needed for subsequent propagation and characterization. In sharp contrast, SIGS can be deployed rapidly simply by designing and preparing exogenous dsRNA, entirely eliminating the need for complex genetic transformation. This characteristic holds significant promise for industrial-scale production and rapid response to emerging pest and disease outbreaks^39^.

The conceptual origins of the SIGS can be traced back two decades. Early studies demonstrated that immersing the model nematode *Caenorhabditis elegans* in dsRNA solution or feeding it with dsRNA-expressing bacteria was sufficient to induce robust gene silencing^40,41^. However, the practical realization of SIGS in crop protection was achieved only in 2016, when the external spraying of dsRNA onto plants was shown to confer resistance against significant fungal pathogens such as Botrytis cinerea and Fusarium graminearum. Since then, the volume of SIGS-related research targeting fungal and other plant pathogens has increased exponentially^42^.

**Fig. 2:**
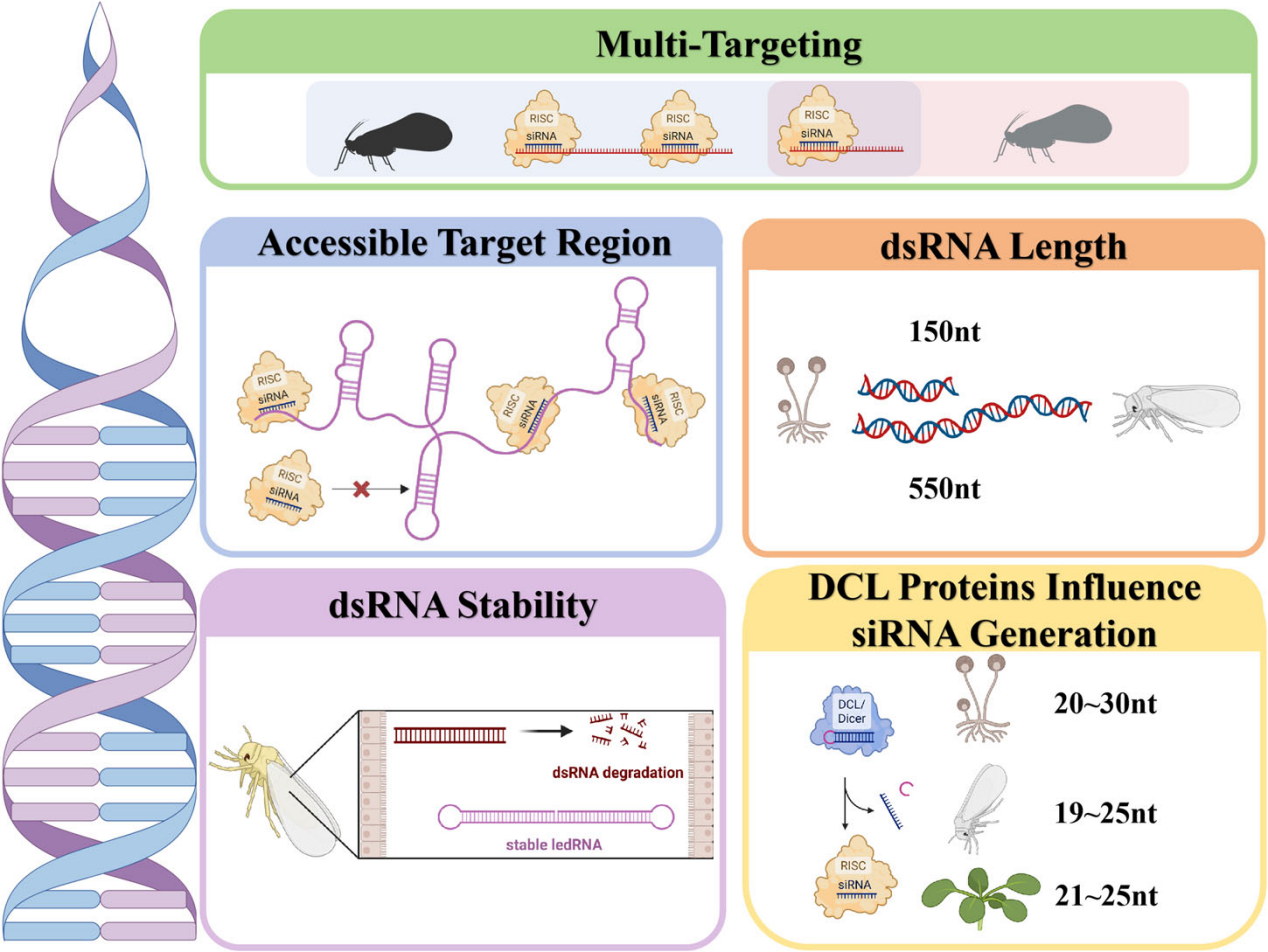
Key factors influencing the efficiency of the SIGS. The diagram illustrates core factors critical to the efficacy of dsRNA/siRNA design: **Multi-Targeting**: Designing siRNAs that target multiple gene loci within the organism enhances RNAi efficiency. **Accessible Target Region**: The secondary structure of target mRNA restricts RISC complex accessibility. Selecting exposed, structurally accessible regions significantly improves silencing efficiency. **dsRNA Length**: The molecular length of dsRNA is a critical factor affecting efficient uptake and processing in different target species. **dsRNA Stability**: Naked linear dsRNA is susceptible to nuclease degradation in the environment or within target organisms. Structural design effectively resists degradation. **DCL Proteins Influence siRNA Generation**: Different organisms possess distinct Dicer/DCL enzymes with varying processing preferences, generating siRNAs of specific size ranges. This requires tailored design optimization for the specific target.

In the SIGS process, once exogenous dsRNA targeting a pathogen gene is applied to a crop, it must first be successfully absorbed by fungal, insect, or plant cells. It is then processed by the host machinery and ultimately assembled into the RISC, which binds to and degrades the complementary mRNA target, thereby achieving effective pest and disease control^43^. Consequently, dsRNA constitutes the core functional element of the entire SIGS system, making the rational design of dsRNA the first critical step in SIGS development^44^. To induce effective gene silencing in the target organism while minimizing potential off-target effects in non-target organisms, the key design elements for dsRNA must be carefully optimized. These elements include target selection, molecular length, secondary structure, the generation of predicted siRNAs, and the preference for guide strand incorporation into the RISC complex (Fig. 2).

### Multiple targeting strategies in dsRNA design

While the majority of SIGS studies demonstrate that targeting a single essential gene can effectively confer pathogen resistance, the degree of disease control often remains highly dependent on the specific target selected. For instance, in the management of Phytophthora infestans, although spraying dsRNA targeting genes like *PiGPB1*, *PiHmp1*, or *PiEndo3* successfully mitigated late blight symptoms, the resulting levels of suppression were markedly inconsistent across different genetic targets^45^. This suggests that relying on a single genetic bottleneck may not always yield stable or sufficient protection. Consequently, targeting multiple transcripts has emerged as a more robust and highly effective strategy for increasing the efficiency of SIGS^46^. Empirical evidence strongly supports this approach: In studies involving barley and *Arabidopsis thaliana*, dsRNA designed to target two *FgCYP51* genes was more effective at inhibiting fungal growth than dsRNA targeting only a single gene, although both strategies successfully conferred resistance to the plants^47^. Furthermore, simultaneously targeting different genes within the same species can also substantially increase resistance. For example, a chimeric DNA construct containing fragments of five different xylanase-encoding genes of Botrytis cinerea (*Bcxyn11A*, *Bcxyn11B*, *Bcxyn11C*, *Bcxyn10A*, and *Bcxyn10B*) was successfully used to achieve a multiplex gene knockdown phenotype in the fungus^48^. Another successful example in Botrytis cinerea involves the use of a single dsRNA to simultaneously target the synthesis genes for three key active transcripts in the fungal sterol biosynthesis pathway, which leads to robust downregulation of all three targeted transcripts. This effectively inhibited Botrytis cinerea germination and growth, thereby reducing gray mold rot^49^.

With rational design, dsRNA can also be engineered to simultaneously target homologous genes across different species^50^. The dsRNAmax tool, for example, was designed specifically to assemble dsRNA sequences that target multiple related RNAs, thus broadening the targeting scope. Its reliability was validated in root-knot nematodes, where the dsRNA was able to suppress multiple root-knot nematode species without affecting non-target nematode species, despite the high homology of their target genes^51^. Another successful example involves researchers designing an artificial optimized dsRNA (iACT) that simultaneously targets the ACT genes of two major piercing-sucking pests—the whitefly (*Bemisia tabaci*) and the green peach aphid (*Myzus persicae*)—by mutually correcting mismatch sites. The iACT retained the conserved regions of the ACT gene in both pests while correcting for species-specific base differences, allowing a single dsRNA to efficiently bind to the ACT mRNAs of both pests. Tobacco expressing iACT was resistant to both whitefly and peach aphids, and the level of resistance was comparable to that of tobacco lines individually expressing *BtACT* or *MpACT*^52^. However, the feasibility of such broad-spectrum strategies is fundamentally constrained by the inherent variability in RNA uptake efficiencies across species. For instance, while pathogens like *Botrytis cinerea* and *Verticillium dahliae* exhibit high dsRNA uptake, others such as Colletotrichum gloeosporioides show no uptake at all, and even the oomycete Phytophthora infestans displays limited and cell-type-specific absorption^53^.

In summary, the rational design of dsRNAs to target multiple sites on a single transcript, multiple transcripts, or even homologous genes across multiple species is a crucial, multifaceted strategy for maximizing silencing efficiency, achieving better control over pest resistance, reducing SIGS production costs, and mitigating the risk of single-target failure.

### Importance of the accessible target region

In dsRNA design, selecting only one or more suitable target genes is insufficient; the specific position of the target within the gene also critically influences silencing efficiency^54^. For example, in rice experiments, dsRNAs targeting different regions within the *Magnaporthe oryzae MoAP1* gene yielded varying results in reducing fungal development and pathogenicity^55^. Similarly, in wheat studies, the silencing efficiency of dsRNAs targeting different regions within the *Chs7*, *Gls*, and *Pkc* genes of F. graminearum also showed distinct differences, thereby strongly supporting the importance of target region accessibility^56^.

Considering the mechanism of RISC-mediated silencing, the internal sequence of the target mRNA needs to interact with the complementary region of the siRNA guide strand^57^. Consequently, this region must be accessible to facilitate the efficient formation of the siRNA/RNA duplex. Studies focusing on perfect RNA hairpin structures revealed that the most tightly folded RNA hairpins were completely resistant to RNAi. However, within a specific range of thermodynamic stability, an inverse relationship exists between the overall stability of the target hairpin structure and RNAi silencing efficiency^58^. Further analysis, on the basis of secondary structure predictions of 103 endogenous human genes, revealed that the free energy cost required to locally unfold the target structure and allow siRNA-guided strand binding is a critical determinant of RNAi activity. Targets requiring a low free energy cost exhibited a significant 40% increase in RNA silencing levels. Although the GC content of a gene also affects its thermodynamic stability and some studies reported a significant negative correlation between GC content and RNAi activity, this correlation is attributable primarily to a high correlation between target site accessibility and GC content. When the influence of target accessibility is controlled, the correlation between the GC content and silencing efficiency nearly disappears entirely^59^. These findings collectively indicate that thermodynamically unstable sequence regions that lack complex secondary structures constitute ideal target locations. Thus, predicting the secondary structure of a target gene via bioinformatics tools is a more crucial design factor than merely considering the GC content.

Notably, in addition to the sequence of the RNA itself, its epigenetic modifications also profoundly affect its secondary structure. For example, structural changes mediated by N6-methyladenosine (m^6^A) lead to greater exposure of UC elements^60^. The methylated groups of m^6^A interfere with the base stacking interactions of the local nucleotides. When m^6^A is present in a double-stranded region, it reduces the local thermal stability of the RNA helix, making it easier to unwind^61^. In contrast, pseudouridine (Ψ) can form water bridges with phosphate groups in the RNA backbone or nearby water molecules, significantly enhancing the local folding stability and thermal stability of the region where Ψ is located. Pseudouridine can stabilize the RNA double-stranded structure when it replaces uracil and forms the Ψ-A, Ψ-G, Ψ-U, and Ψ-C pairs^62^.

Finally, unlike gene editing, the primary target of RNAi is mRNA. This means that dsRNA targeting intron regions will not result in gene silencing, and the target region must therefore be located exclusively within the exon boundaries of the gene^63^. Moreover, the 5’ untranslated region (5’ UTR) and 3’ untranslated region (3’ UTR) of the mRNA represent excellent targeting options, as these regions typically exhibit less complex secondary structures and are therefore often more susceptible to the RNAi effect^64^.

### dsRNA length: a receptor-specific critical parameter

The length of the dsRNA molecule is a critical parameter in dsRNA design because it influences the recipient’s ability to internalize and process the molecule. Furthermore, longer dsRNA generates a greater variety and number of siRNAs after cleavage by Dicer-like (DCL) proteins. However, dedicated research focusing specifically on dsRNA length optimization in SIGS remains relatively limited to date.

Intuitively, longer dsRNA should translate to stronger silencing owing to the generation of a wider array of siRNAs. This theoretical benefit, however, does not necessarily lead to more potent disease control, as longer dsRNA may be constrained by the recipient’s uptake capacity. The evidence from studies on fungi is mixed and suggests limitations. In one study, the silencing efficiency of dsRNA actually decreased as the length increased from 220 nt to 1500 nt^65^. This reduced efficiency may be caused by limitations in pathogen uptake, as 1500 nt dsRNA failed to silence *F. graminearum* when applied in liquid culture, indicating that the fungus struggled to absorb that length directly. Notably, when ≥1500 nt dsRNA was sprayed onto barley leaves, it successfully reduced the pathogenicity of *F. graminearum*. This success is likely because the host plant preprocessed the molecule into smaller dsRNA or siRNA fragments that the fungus could then internalize. Furthermore, certain fungi, such as those in the *Phytophthora* genus, do not appear to take up longer amounts of dsRNA directly from the environment. Nevertheless, SIGS using short dsRNAs remains viable for these pathogens, with studies showing that 30–75 bp dsRNAs can completely inhibit spore germination, achieving significantly greater efficiency than 21–25 nt dsRNAs^53^. In summary, while longer dsRNA can theoretically increase silencing efficiency, fungal uptake capacity is a more critical limiting factor.

Compared with fungi, insects appear to exhibit a superior ability to internalize long dsRNA. In vitro experiments on the midgut of *Tribolium castaneum* suggested that SIGS silencing efficiency is positively correlated with dsRNA length^66^. Moreover, in the Colorado potato beetle, dsRNA lengths ranging widely from 141 bp to 1506 bp have been successfully applied^67^. Conversely, dsRNAs shorter than 60 bp exhibit poor silencing efficacy in insects, with 21 bp dsRNAs showing almost no effect^67,68^.

Overall, the optimal dsRNA length appears to be receptor-specific, which is determined by the interplay between the characteristics of DCL proteins and the receptor’s environmental uptake capacity. Additionally, if the dsRNA is first absorbed by the host plant and subsequently transferred to the target recipient, the plant’s own uptake and DCL preferences may also influence the optimal length^69^. Consequently, accurately predicting the optimal dsRNA length remains challenging. Therefore, it is necessary to test different dsRNA lengths when developing new SIGS strategies. In existing research, dsRNA sizes within the range of 150–550 nt are the most prevalent, making this range a practical starting point for design^67,68^. Importantly, the results obtained from HIGS may differ from those obtained from SIGS, likely because of the plant’s *in planta* dsRNA processing, thus limiting their direct utility as a reference for appropriate dsRNA length in SIGS^70^.

### Optimizing the sequence to enhance dsRNA stability

During the SIGS process, dsRNA must remain stable in the external environment for an extended period to allow for absorption by the recipient organism or plant cells. Additionally, when targeting insect pests, the dsRNA must be robust enough to withstand the highly degradative environment of the insect gut^71^. Therefore, dsRNA stability is a crucial factor influencing overall silencing efficiency. Rational optimization of the dsRNA sequence can inherently increase its thermodynamic stability and consequently reduce its rate of degradation^72^.

One of the most direct and effective approaches to improve stability is to engineer the dsRNA to form a stable secondary structure, such as the loop-end-enhanced dsRNA (ledRNA) structure. The ledRNA molecule possesses a unique dumbbell-shaped structure, consisting of a long dsRNA stem flanked by single-stranded loops, with a nick site at the dsRNA stem. Compared with that of conventional linear dsRNA, the distinctive closed-loop structure of ledRNA significantly increases the stability of the RNA molecule. When ledRNAs were locally applied to leaves, cotyledons, meristems, or roots, their accumulation levels in both treated tissues and untreated, distant tissues (such as adjacent leaf regions and far-off cotyledons and roots) were greater than those of traditional hairpin RNAs (hpRNAs). These findings suggest that ledRNAs offer superior stability and more effective plant uptake and mobility than traditional RNAi molecules do^73^. Comparative studies targeting the brown planthopper (*Nilaparvata lugens*) revealed that, compared with conventional dsRNA and hairpin dsRNA, ledRNA induced greater gene silencing when it was orally fed to insects. However, under microinjection and immersion treatments, where the gut barrier is bypassed, no significant difference in silencing efficiency was observed among the three RNA types. This strongly suggests that the closed-loop structure of ledRNAs primarily enhances the stability of the dsRNA against degradation within the insect gut environment^74^.

In conclusion, ledRNA represents a simple and highly effective strategy for increasing dsRNA stability, particularly when countering degradation in the insect gut. Consequently, utilizing ledRNA structural design is an excellent optimization choice when the target recipient is an insect pest.

### Influence of DCL proteins on siRNA generation

Since gene silencing efficiency is dependent on the resulting siRNA sequence, and the siRNA is, in turn, determined by the cleavage length and mechanism of the DCL or Dicer proteins, a deep understanding of DCL or Dicer, particularly the length of the siRNAs they produce, is critical for effective dsRNA design^75^.

Unfortunately, in fungi, the siRNA lengths generated by DCL cleavage exhibit both conservation and diversity, typically ranging between 20 and 30 nt^76–81^. Even within a well-characterized model fungus such as *Neurospora crassa*, multiple siRNAs are produced; in addition to the dominant 25 nt siRNAs, *N. crassa* also generates shorter siRNAs of approximately 21–23 nt, which are often associated with DNA damage^82,83^.

In contrast to the complexity observed in fungi, the siRNA processing pathway in insects is more highly conserved. In most insect species, including major agricultural pests, the dsRNA precursor molecule is recognized by Dicer-2 and precisely cleaved into siRNA fragments, typically between $19$ nt and $25$ nt^84–88^. Dicer-2 performs repetitive cleavage at fixed distances, ensuring that siRNAs have consistent length and polarity for effective loading into the RISC to mediate target mRNA degradation^89,90^. Given the fixed nature of siRNA length in insects, it is possible to significantly reduce environmental risk by designing dsRNA sequences whose resulting siRNAs do not share sufficiently high homology with non-target species sequences.

Fungi and insects sometimes also take in miRNAs and siRNAs processed by plants^91^. For example, *Arabidopsis thaliana* has four DCLs, which process dsRNA and form 21 nt miRNAs, 22 nt siRNAs, 24 nt hr-siRNAs, and 21 nt ta-siRNAs^92^. Among these genes, DCL2 and DCL4 are considered to play a core role in virus defense, but the latest research shows that DCL3 is necessary in the defense against cucumber mosaic virus^93,94^. Moreover, the number of members of the DCL family varies among different plant species. For example, there are 7 DCL family members in tomato, whereas there are 8 in rice. Therefore, considering the specificity of the plant host when designing dsRNA is also meaningful.

In summary, DCL proteins in fungi and Dicer proteins in insects differ in their siRNA product preferences: insect Dicer function is highly conserved, whereas fungal DCL is more diverse, suggesting a greater need for research to characterize fungal DCL activity. Understanding how DCL or Dicer processes the dsRNA substrate into siRNA products is instrumental in designing dsRNA molecules that can be effectively processed into siRNAs by the target pathogen’s DCL or Dicer. For example, dsRNA can be designed to be processed by specific DCL proteins to yield siRNAs within the commonly reported length range of 21--25 nt for both fungi and insects. Alternatively, the dsRNA sequence can be designed to ensure that the resulting siRNAs do not share sufficiently high homology with non-target species sequences, thereby significantly minimizing environmental risk.

In summary, multiple dimensions have been identified for designing efficient and stable dsRNA for SIGS. However, they must be harmonized with downstream production and delivery constraints to be truly viable. For instance, although longer dsRNAs (>500 nt) may theoretically enhance silencing potency, they often compromise bacterial expression yields due to transcriptional collisions or plasmid instability, and complicate uniform loading into nanocarriers. Currently, the empirical data bridging specific dsRNA structural traits with large-scale manufacturing and nano-delivery remains insufficient. Consequently, establishing a holistic design framework that accounts for these downstream bottlenecks remains a significant challenge for the field.

### Production, optimization, and nanomaterial-mediated protection of dsRNA for SIGS

The production cost and stability of dsRNA are pivotal factors determining the feasibility of implementing SIGS in practical agricultural production. Currently, dsRNA can be obtained through three main methods: chemical synthesis, in vitro transcription (IVT), and in vivo production by engineered microorganisms^95,96^. Traditional chemical synthesis typically involves the direct synthesis of single RNA strands via solid-phase synthesis techniques, such as the phosphoramidite method, followed by annealing to form a double-stranded structure. However, this method is characterized by a high cost, and its yield decreases exponentially as the RNA length increases^97,98^.

In vitro transcription (IVT) refers to synthesis outside the cell via an RNA polymerase and a DNA template. The predominant IVT methods utilize T7 RNA polymerase and a DNA template to transcribe sense and antisense strands separately, which are then annealed to form dsRNAs. Alternatively, a DNA template with a loop sequence in the middle and inverted complementary sequences at the ends is transcribed to form partially complementary hairpin RNA (hpRNA) with a stem‒loop structure^96^. In vivo synthesis, conversely, involves the use of modified engineered microorganisms to complete a process analogous to IVT, which is based on exogenous DNA inside the microbial cell. The mainstream approaches here also involve either the annealing of sense and antisense strands or the self-annealing of a single hairpin strand.

### Production of dsRNA: current methods, comparison, and future directions

Although the methods described above provide a solid technical foundation for dsRNA production, their practical application in agriculture remains limited. It is estimated that 2 to 10 grams of dsRNA per hectare are required for effective field spray control of major agricultural pests and diseases. Consequently, the key to successful commercialization of SIGS lies in the ability to produce high-quality dsRNA at low cost and on a large scale.

Current production methods vary significantly in cost and scalability. Chemical synthesis, while highly precise, is prohibitively expensive for long dsRNA sequences. Conventional IVT is widely used in laboratory settings; however, the high cost of nucleotide substrates, enzymes, and purification equipment results in pilot-scale production costs of approximately $50–$60 per gram, making large-scale field application economically unfeasible^99^. Microbial fermentation using engineered microorganisms has emerged as a more cost-effective alternative. Systems based on RNase III-deficient *E. coli* or *Corynebacterium glutamicum* can achieve yields of 0.3–1.0 g/L, with pilot-scale costs potentially dropping to $2–$5 per gram^100^. These platforms benefit from mature fermentation infrastructure and relatively low raw material costs. Nevertheless, challenges such as plasmid instability, batch-to-batch variability, and stringent downstream purification requirements remain.

Cell-free synthesis platforms have demonstrated strong potential for agricultural scale-up. GreenLight Biosciences has successfully commercialized its proprietary cell-free system, which utilizes cell extracts, enzymes, and short DNA templates to produce Calantha™ (Ledprona)—the first sprayable dsRNA bio-insecticide approved by the U.S. Environmental Protection Agency (EPA)^101^. This platform reportedly achieves production costs approaching or below $0.5–$1 per gram at commercial scale, while offering high sequence flexibility and minimal infrastructure requirements^102^.

It is important to interpret these cost estimates with caution. The reported figures are typically based on optimized pilot or industrial processes and may not fully account for additional expenses such as downstream purification, nano-carrier formulation, quality control, and regulatory compliance. Actual costs can vary substantially depending on production scale, geographic location, infrastructure availability, energy prices, and the specific dsRNA sequence. Furthermore, issues including plasmid instability, inducer toxicity, and batch-to-batch variability in microbial systems still require further optimization to ensure consistent industrial production.

In summary, no single production platform is perfect. However, it is clear that microbial fermentation and cell-free synthesis will be the dominant systems for future agricultural applications of SIGS, replacing chemical synthesis. At present, microbial fermentation offers the most practical route for near-term scale-up, while cell-free systems hold greater promise for long-term cost reduction and regulatory acceptance. Hybrid approaches that combine microbial enzyme production with cell-free assembly are likely to emerge as the optimal solution.

In practice, platform selection should be guided by comprehensive techno-economic analyses that consider not only raw production costs but also the total cost within real-world agricultural supply chains. Therefore, advancing SIGS requires priority focus on optimizing engineered microorganisms, expression vectors, and dsRNA protection strategies to enable lower-cost and more efficient field applications.

### Engineered microorganisms for dsRNA synthesis

**Fig. 3:**
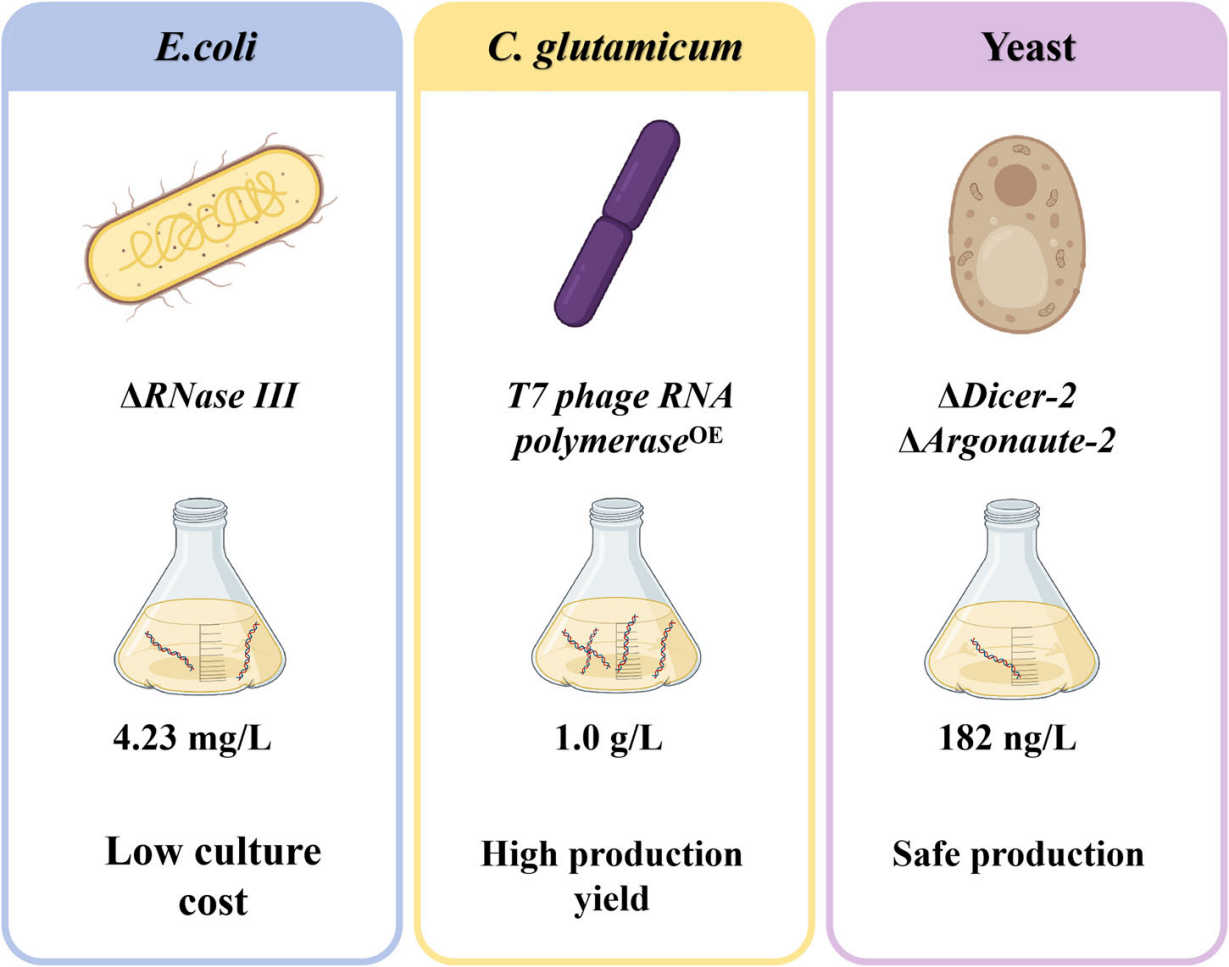
Increasing dsRNA production via engineered microorganisms. The schematic illustrates genetically modified microbial host systems and their engineering strategies for enhancing dsRNA yields: ***E. coli***: Engineered via knockout of the RNase III gene to inhibit endogenous dsRNA degradation. The fermentation yield is 4.23 mg/L, with the primary advantage of low culture costs. ***C. glutamicum***: Engineered through overexpression of T7 phage RNA polymerase to establish a highly efficient transcription system. It achieves a high yield of 1.0 g/L, demonstrating superior production yields. **Yeast**: Modified via dual knockout of *Dicer-2* and *Argonaute-2* to block the host’s native RNAi pathway and prevent dsRNA processing. The yield is approximately 182 ng/L, and its key advantage lies in its generally recognized as safe status.

The use of recombinant microorganisms as fermentation factories for dsRNA production is a highly cost-effective and efficient strategy, as microbes are easy to handle, grow quickly, and stably retain plasmids^103^. Currently, *Escherichia coli*, yeast, *Corynebacterium glutamicum*, and bacteriophages have been successfully employed for dsRNA production (Fig. 3).

*E. coli* is one of the most important model organisms and is widely developed as an engineered strain for the production of various products because of its clear genetic background, rich genetic operating system, rapid growth, and low culture cost. The HT115 (DE3) system is the most commonly used system for dsRNA production in laboratories. The HT115 (DE3) strain has genes encoding the ribonucleases RNase D and RNase E, as well as the gene encoding the dsRNA-specific endonuclease RNase III, which is inactivated, significantly increasing the strain’s capacity to synthesize and accumulate dsRNA^41,104,105^. Multiple teams have further refined the strain, for example, by mutating the *rnc* and *lacY* genes to improve the uniformity of its response to the IPTG inducer^106^. Furthermore, significant results have been achieved through the development of novel strains, such as the RNase III deletion strain pET28-BL21 (DE3), constructed on the basis of the pBL21 (DE3) strain, which achieved a final dsRNA yield of 4.23 mg/L under identical conditions—three times the yield of L4440-HT115 (DE3)^107^.

*Corynebacterium glutamicum* is a primary strain used in amino acid production, and recent studies have revealed its potential to produce large amounts of RNA^108^. By constructing a system that utilizes the strong F1 promoter convergence transcription system of the bacteriophage BFK20 and introduces the RNA-binding U1A protein, researchers successfully enabled *C. glutamicum* to achieve a dsRNA yield of 300 mg/L^109^. In subsequent studies, by employing a plasmid (pPK4H1) with an approximate copy number of 800, T7 phage RNA polymerase, and a terminator, the yield was further increased, reaching 1.0 g/L dsRNA in a 0.3 L fermenter^110^.

Yeast is a safe production strain commonly used in fermentation, featuring a clear genetic background, a rich genetic operating system, and established fermentation processes. Although some reports suggest that the expression product of the *rnt1* gene in yeast exhibits dsRNA cleavage activity, yeast generally lacks the two core RNAi pathway genes *Dicer-2* and *Argonaute-2*, making it a potential cell factory for dsRNA accumulation^111^. In terms of application, a study utilized *Yarrowia lipolytica* to produce dsRNA aimed at suppressing white spot syndrome virus in shrimp. However, the yield of this system was lower than that of the other systems, reaching only 182 ng/L^112^.

Phages are viruses that infect bacteria and are capable of replicating and producing large amounts of genetic material inside the host cell. In 1973, the bacteriophage Φ6 was isolated from *Pseudomonas syringae*; since its genetic material is dsRNA, it can be utilized for dsRNA synthesis^113^. Currently, researchers have developed a dsRNA production system based on Φ6, where the produced dsRNA is encapsulated in a protective capsid, effectively preventing degradation by endogenous RNases. Small-scale production and large-scale fermenter experiments confirmed that when this three-plasmid system cooperated within the host cell, highly specific dsRNA was produced at a yield of approximately 1.6 mg/g wet cell weight^114^. In plant research, this system was used to confer tobacco mosaic virus (TMV) resistance, generating dsRNA longer than 2600 bp and simultaneously producing two different dsRNA sequences. Mechanical inoculation or spraying of TMV dsRNA purified from inoculated *P. syringae* confers resistance to TMV in tobacco plants^115,116^.

Compared with wild-type strains, the engineered strains reported to date have achieved highly efficient dsRNA production through modifications of key genes and regulatory elements in expression plasmids. With continuous technological advancements, more microbial species and more efficient regulatory elements are likely to be discovered, providing more options and further increasing yields for large-scale dsRNA production, thereby driving its application in agriculture and medicine.

### Vector selection and optimization

**Fig. 4:**
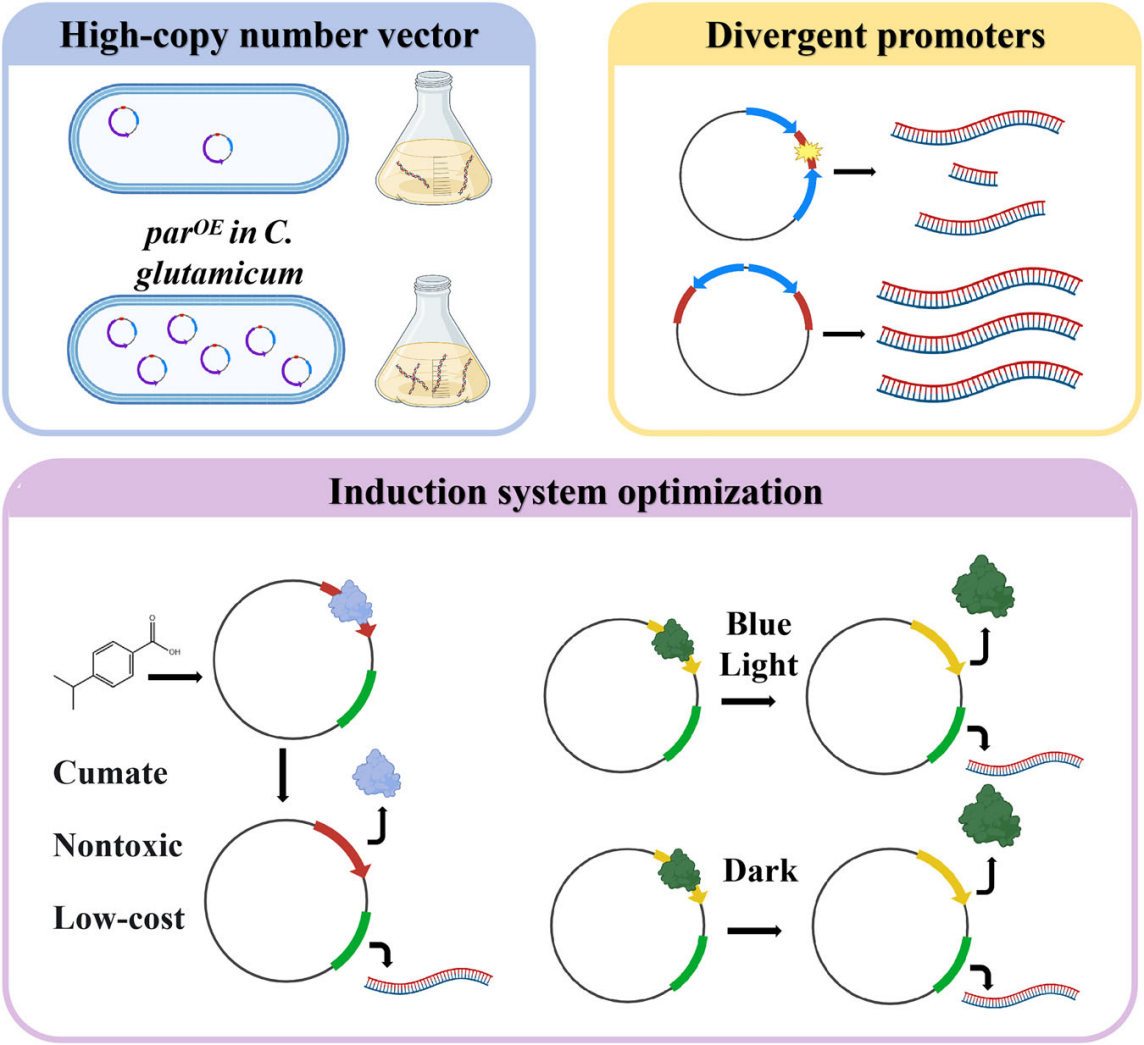
Optimizing dsRNA production through vector engineering. The schematic illustrates vector optimization strategies aimed at increasing dsRNA yields in microbial systems: **High-copy number vector**: By overexpressing the plasmid partitioning gene *par in C. glutamicum*, the plasmid copy number within the host cell is significantly increased, thereby elevating the availability of DNA templates for dsRNA synthesis. **Divergent promoters**: Utilizing a pair of opposing promoters to simultaneously transcribe both strands of the DNA duplex, enabling highly efficient and coordinated production of dsRNA from a single vector. **Induction system optimization**: The left panel shows the Cumate-inducible system, characterized by being nontoxic and low-cost. The right panel illustrates a light-inducible system, utilizing light-sensitive transcription factors to achieve environmentally friendly, chemical-free induction of dsRNA expression.

The expression vector is another critical factor determining dsRNA production efficiency. Strategies such as optimizing the intracellular plasmid copy number, developing novel nontoxic induction systems, and refining T7 promoter-driven transcription elements can significantly increase dsRNA production yields (Fig. 4).

The most direct strategy for increasing dsRNA yield is to increase the intracellular plasmid copy number, which is primarily determined by its origin of replication (*ori*). For example, using a high copy number derived from the pUC series typically ensures that high levels of plasmid copies are maintained within the cell^117^. In a study on dsRNA production in *Corynebacterium glutamicum*, changing the plasmid from one with approximately 300 copies to one with approximately 800 copies increased the dsRNA yield from 75 mg/L to 150 mg/L (Hashiro et al., 2021). However, the same study indicated poor stability of the dsRNA production plasmid in *C. glutamicum*, with a plasmid retention rate less than 20%. This suggests that replacing the terminator with a more efficient terminator or introducing a plasmid partition system (such as the *par* gene) would help increase plasmid stability and further promote dsRNA accumulation^118^.

With respect to the precise regulation of transcription elements, while the T7 phage RNA polymerase (T7 RNAP) exhibits extremely high transcriptional activity, the opposing polymerases undergo transcription collision when simultaneously synthesizing RNA from two complementary strands, which inhibits dsRNA synthesis efficiency. To resolve this issue, a crucial structural design employs divergent promoters, where two T7 promoters are arranged back-to-back to drive transcription, thus avoiding a head-on collision between polymerases on the template. Research has shown that this design can increase the yield by 2.1-fold for dsRNAs larger than 400 base pairs^96^. Furthermore, using multiple high-efficiency transcription terminators in tandem (e.g., the T7 Φ terminator) at the end of the transcription unit is also vital. This effectively prevents T7 RNAP read-through, ensuring that transcription stops accurately, thereby reducing the formation of dsRNA polymers and increasing product quality and purity.

The selection of the induction system is also central to vector optimization. The conventional IPTG induction method is restricted in large-scale industrial fermentation because of its cytotoxicity and cost, prompting researchers to develop novel nontoxic inducible agents or environmentally condition-responsive systems. In the context of nontoxic chemical inducers, in addition to traditional natural metabolite-based systems such as arabinose or rhamnose, recent studies have developed novel inducible gene expression systems for *Bacillus subtilis* and *Bacillus megaterium* that use para-isopropylbenzoic acid (cumate) as inducers^119–121^. This system fuses the strong constitutive promoter Pveg from *B. subtilis* with regulatory elements from *Pseudomonas putida*, using the CymR repressor protein to inhibit transcription in the absence of the inducer. Upon cumate addition, CymR dissociates, increasing repression and initiating transcription. By regulating the strong Pveg promoter with the CymR repressor, this system achieves induction that is nontoxic to the host, low-cost, and carbon source independent. Moreover, induction systems based on environmental physical signals also show promise. The most traditional of these uses the λ phage cI857 temperature-sensitive repressor to achieve thermocontrolled expression^122^. In the latest research, the LacI repressor protein from the *lac* operon was engineered for photocontrol by introducing the blue-light-responsive element AsLOV2 domain. This created blue-light/dark-responsive repressor mutants (OptoLacIL and OptoLacItextD), which were used to construct two light-controlled *E. coli* gene expression systems: *OptoE.coliLight* and *OptoE.coliDark*. The *OptoE.coliDark* system (in which light inhibits expression and darkness initiates expression) has been applied for protein production and metabolic pathway regulation. Overall, nontoxic promoters and environmental induction strategies hold immense potential as alternatives to IPTG in dsRNA production, but the actual increase in dsRNA yield from these alternatives still requires further in-depth study and validation^123^.

### Protection of dsRNA using nanomaterials

In practical applications, dsRNA has consistently faced challenges regarding its susceptibility to degradation, limited duration of protection, and insufficient absorption by target cells. Furthermore, dsRNA must successfully penetrate the plant cuticle, cell wall, and plasma membrane to enter plant cells^124^. The plant cell wall is a major barrier to nucleic acid delivery, typically restricting the passage of nucleic acid molecules less than 20 nanometers in diameter. To control fungal or oomycete infections, sprayed RNA does not need to enter the plant interior; it can remain on the plant surface and be absorbed directly by the pathogen. However, when targeting insects, dsRNA must be robust enough to withstand the digestive action of the insect gut^125^. In recent years, the use of nanomaterials has shown great potential in mitigating dsRNA degradation and enhancing dsRNA delivery efficiency within plants or pests.

**Fig. 5:**
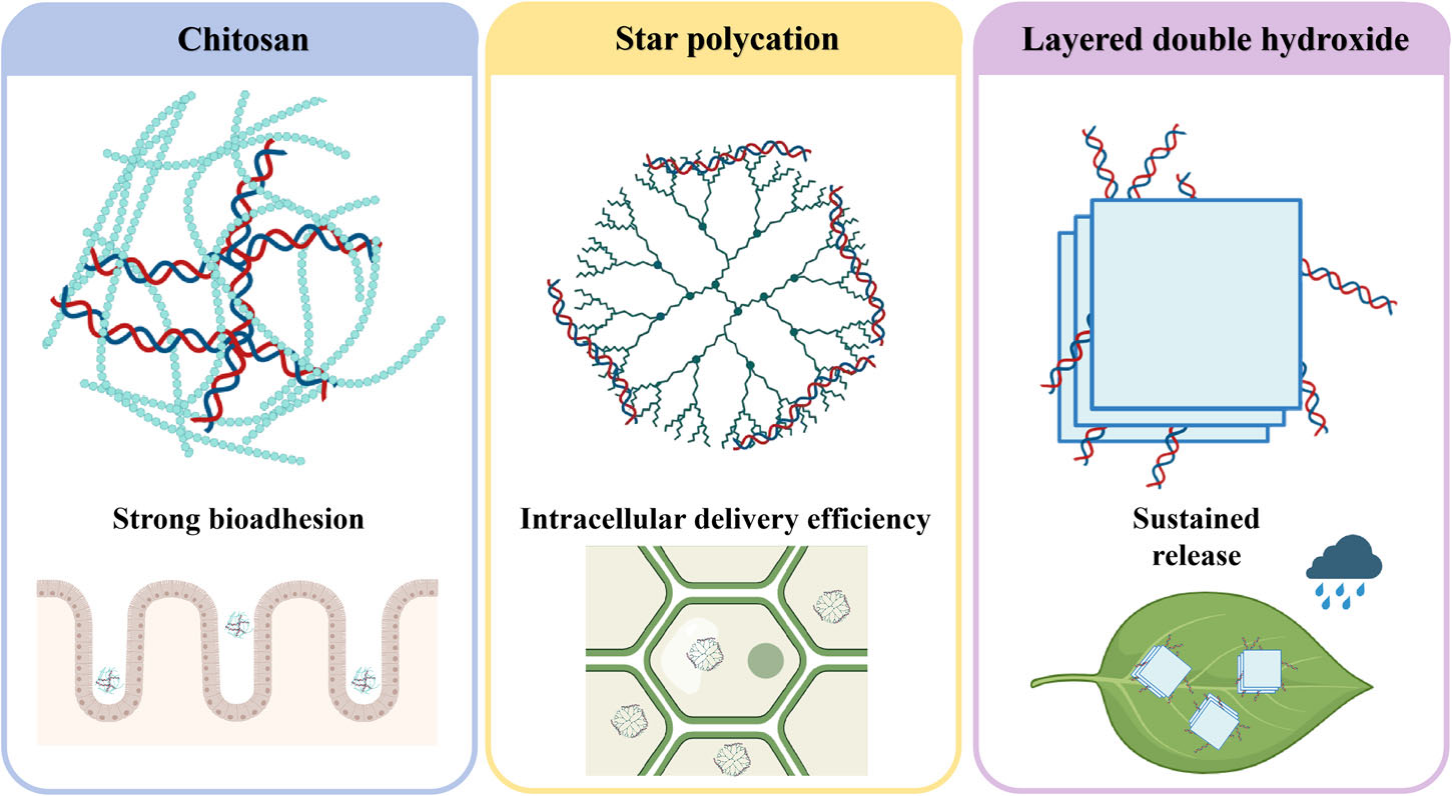
Nanoparticle-mediated dsRNA delivery systems and their functional strengths. The schematic illustrates three nanocarrier strategies for protecting and delivering dsRNA, along with their core functional characteristics: **Chitosan**: A natural cationic polysaccharide that encapsulates negatively charged dsRNA via electrostatic interactions to form nanocomplexes. Its primary advantage lies in strong bioadhesion to facilitate intestinal uptake. **Star polycation**: A highly branched star-shaped polymer that binds dsRNA densely through multivalent positive charges. Its core strength is high intracellular delivery efficiency for enhanced membrane translocation. **Layered double hydroxide**: Intercalates dsRNA within layers, offering sustained release and resistance to rain wash-off, making it suitable for foliar application and long-lasting protection.

Despite the existence of multiple classes of nanomaterials for dsRNA delivery, including carbon-based nanomaterials, metal-based nanomaterials, nanoliposomes, and peptide-based nanomaterials, polymer-based nanomaterials are currently the most widely applied materials in plant SIGS (Fig. 5).

Chitosan (CS), a deacetylated derivative of chitin extracted from crustaceans, is widely recognized as a biocompatible, adhesive, and penetration-enhancing drug delivery system because of its small size and positive charge^126^. By targeting the key pathogenicity genes *RsAGO1* and *RsAGO2* of *R. solani*, the chitosan/star polycation complex (CSC) was proven to increase dsRNA stability and improve resistance against nucleases. Both CSC and CS effectively increased the efficiency of pathogen uptake of dsRNA, and CSC could extend the duration of the protective effect of dsRNA for up to 20 days^127^. CS is more extensively applied in insect control. By complexing dsRNA with CS, the dsRNA-CS complex was protected from endonuclease degradation in the cell culture medium, hemolymph, and midgut lumen contents of *Sesamia inferens* (Asian corn borer) larvae. This complex significantly reduces the expression of the endogenous *iap* gene in larvae, resulting in growth retardation and mortality^128^. In *Chilo suppressalis* (striped rice borer), a major rice pest, the application of CS complexed with dsRNA, which targets the *G3PDH* gene, effectively silences *G3PDH* and causes the death of *C. suppressalis*^129^. Furthermore, chitosan nanoparticles (CNPs) effectively mediated the delivery of specific dsRNAs targeting the *JHAMT* and *ACHE* genes in *Helicoverpa armigera* larvae. The CNPs-dsRNA complexes remained stable on the leaf surface for 5 days, and ingestion along with the leaf effectively silenced the *JHAMT* and *ACHE* genes in the larvae, inhibiting the corresponding enzyme activities and resulting in 100% insect mortality^130^.

Dendrimers are highly branched, structurally precise polymers whose cationic surface or internal cavities allow for the efficient encapsulation of bioactive molecules^131^. Star polycation (SPc), a type of dendrimer, is a low-cost dsRNA nanocarrier with excellent intracellular delivery efficiency and biodegradability^132^. In combating *Phytophthora infestans*, the causative agent of potato late blight, SPc-dsRNA overcomes the delivery bottleneck of *P. infestans* for dsRNA, extending the RNAi protection window^133^. SPc is also widely used in insect control. By targeting the methoxyfenozide tolerance protein (*Met*)/Tai protein (*Tai*), which are components of the contacticide receptor in *Adelphocoris suturalis* larvae, the SPc-dsRNA complex, when applied via a topical spray system, resulted in RNAi knockdown efficiencies ranging from 39% to 58% and led to distinct abnormal ovarian development^134^. In *Aphis gossypii* (cotton aphid), SPc-dsRNAs targeting the chitin pathway genes *AgCHS2* and *AgHK2* significantly increased the mortality of *A. gossypii* under both laboratory and greenhouse conditions^135^. SPc-dsRNA-based SIGS targeting *DcCP8* also significantly increased the mortality of *Diaphorina citri* (Asian citrus psyllid)^136^.

Additionally, layered double hydroxide nanosheets (LDHs) are positively charged, ionic layered inorganic materials commonly used in plant SIGS. LDH nanosheets form neutral complexes when bound to negatively charged biomolecules. These complexes can efficiently penetrate cell membranes via nonendocytic pathways, suggesting significant potential for gene delivery^137^. Furthermore, the interaction between LDH nanosheets and surrounding ions has the potential to increase dsRNA stability and release^138^. Consequently, LDH nanosheets are highly effective nanocapsulation materials for SIGS. LDH nanosheets were first used as gene delivery vectors to manage plant viruses, specifically pepper mild motor virus (PMMoV) and cucumber mosaic virus (CMV), owing to their efficient delivery of genes into plant cells. Once loaded onto LDH, the dsRNA does not detach, allows for sustained release, and remains detectable on sprayed leaves for up to 30 days after spraying. A single spray of dsRNA-loaded LDH provided plants with at least 20 days of viral protection when challenged, including newly emerged, unsprayed leaves^139^. In addition to being used for viral control, LDH nanosheets have been used for dsRNA delivery to manage fungal diseases. In combating *Botrytis cinerea*, incorporating dsRNA into LDH nanoclays resulted in the LDH‒dsRNA complex retaining its efficacy during six weeks of cold storage and significantly reducing gray mold development after long-term storage. The study also revealed that increased CO_2_ concentration and humidity accelerated LDH degradation and promoted dsRNA release from the complex, enabling controllable SIGS regulation^140^. In insects, LDH nanosheets have also shown promise. By modifying LDH with fatty acids to create an amphiphilic, surfactant-like LDH (LDHS), good leaf adhesion, transportation capacity, and excellent biocompatibility were achieved. When loaded with dsRNA, LDHS was demonstrated to increase the uptake of dsRNA by the brown planthopper (*Nilaparvata lugens*) via direct spray application. The amount of dsRNA delivered was 2.5 times greater than that of hydrophilic LDH with an adjuvant, leading to higher mortality rates in phytophagous insects^141^.

The selection of an optimal nanocarrier is a sophistic amentally governed by the structural morphology and electrochemical profile of the dsRNA. Specifically, the length of the dsRNA and its charge distribution play decisive roles in determining the success of the delivery system. Shorter dsRNA molecules, particularly those engineered to target highly accessible regions, demonstrate significantly higher loading efficiency and more robust intercalation within LDH nanosheets^142^. Furthermore, to ensure high payload capacity and prevent competitive binding, rigorous downstream purification is often essential before the complexation process can proceed, ensuring the resulting nano-assemblies are both stable and biologically potent^143^.

In conclusion, nanoparticle-mediated RNAi enhances nucleic acid stability and delivery efficiency, indirectly lowering the cost of the practical application of RNAi. It protects dsRNA, reduces the rate of nucleic acid degradation, and simultaneously improves dsRNA penetration, ultimately increasing the efficiency of gene silencing. Nevertheless, the performance of various nanomaterials is highly contingent upon the specific species and application environment. This inherent variability necessitates the screening of multiple nanomaterial candidates to guarantee reliable SIGS-mediated protection^144,145^.

### Biosafety, regulatory considerations, and environmental risk assessment

Although spray-induced gene silencing (SIGS) is recognized as a highly specific alternative to conventional chemical pesticides, its commercialization still requires biosafety evaluations.

Well-curated dsRNAs minimize homology to non-target genomes through stringent bioinformatics filtering. However, off-target silencing remains a possibility if siRNAs share sufficient matches with transcripts in phylogenetically related species. Exposure pathways for non-target organisms include direct contact, trophic transfer, and indirect movement through soil or aquatic systems. Closely related taxa are most susceptible; distant lineages often exhibit reduced sensitivity due to divergent RNAi machinery or physiological uptake barriers. Consequently, a tiered risk assessment is necessary^146^. Furthermore, while multi-target designs enhance efficacy, they necessitate broader off-target screening. Overall, current evidence suggests that risks are minimal when sequences are carefully selected.

Nanomaterials significantly enhance dsRNA delivery and protection but introduce additional risks. Carriers such as chitosan, SPc, and LDH generally exhibit favorable biodegradability and low acute toxicity at field-relevant dosages. Nevertheless, they may extend the environmental persistence of dsRNA, facilitate unintended uptake in NTOs, or release specific carrier components^147^. Therefore, ecotoxicological testing must be integrated into the development process. Despite existing data gaps regarding long-term field behavior, current risks appear manageable at proposed application rates.

Human exposure is primarily restricted to occupational routes during spray application, with negligible dietary risk. Mammals possess effective biological barriers to dsRNA activity, including salivary and gastrointestinal nucleases and highly acidic gastric environments. Currently approved products are classified as practically non-toxic. While nanocarriers may alter bioavailability, necessitating specific gastric stability data^148^. Therefore, appropriate personal protective equipment and low-drift application techniques will further minimize risks.

In the United States, the EPA regulates externally applied dsRNA products as biochemical pesticides under FIFRA and FFDCA. One example is Calantha™, the first sprayable dsRNA insecticide registered in 2023–2024 for control of Colorado potato beetle on potatoes^101^. It received a conditional registration supported by data showing rapid degradation, negligible residues, and low risk to human health and the environment.

The European Union often applies stricter scrutiny, sometimes requiring extended ecological data. OECD guidance documents on environmental and human health risk assessment for externally applied dsRNA pesticides provide harmonized frameworks for data requirements and exposure pathways^148,149^. Key commercialization challenges include the lack of fully standardized protocols for nano-formulated products, high assessment costs, lab-to-field consistency, public perception of SIGS and resistance management. Transparent, science-based evaluations are essential for building regulatory confidence and acceptance.

In summary, SIGS offers significant specificity and reduced environmental load compared with broad-spectrum chemicals. Through interdisciplinary collaboration among molecular biologists, toxicologists, agronomists, and regulators, RNAi technology can become a sustainable tool for crop protection.

### Outlook

The application of RNAi technology in agricultural pest and pathogen control has vast potential, yet its large-scale commercialization and sustainable development still hinge on overcoming a series of critical technological bottlenecks.

The main objective is the continuous optimization of dsRNA design and the enhancement of target prediction accuracy. Contrary to earlier assumptions, emerging evidence suggests that dsRNA length is not the definitive determinant of resistance. Instead, uptake efficiency and environmental context often outweigh intrinsic design parameters. These data must be integrated into efficient, automated design platforms to develop more potent dsRNA molecules.

The transition from laboratory-scale yields to industrial-scale production remains a significant hurdle. Many laboratory-proven yields have yet to translate reliably to commercial manufacturing. Although engineered microbial and cell-free systems have reduced costs, scaling is hampered by plasmid instability and inducer toxicity during fermentation. Achieving consistent, low-energy, and standardized processes capable of meeting the agricultural demand for dsRNA requires further optimization.

Most critically, the development of efficient and safe nanodelivery systems remains a key limitation. The performance of nanocarriers under field conditions often is inferior to results observed in controlled laboratory trials. Formulations that appear stable in vitro tend to degrade rapidly when exposed to environmental stressors such as UV radiation, precipitation, and temperature fluctuations. Furthermore, comprehensive environmental and human safety assessments are still lacking for many formulations; the biosafety risks associated with long-term soil accumulation, potential impacts on non-target organisms (e.g., beneficial microbiota and pollinators), and unintended off-target gene silencing remain largely unknown. Addressing these obstacles through multi-location field trials and advanced nanocarriers is essential for transitioning RNAi technology from proof-of-concept studies to large-scale agricultural adoption.

Therefore, the combination of multifactorial dsRNA design, scalable synthesis, and precision delivery will define the future commercial viability of SIGS-based solutions.

### Grants and funding

This work was supported by the Science Foundation for Outstanding Young People of Xinjiang (No. 2024D01E36).

## Supplementary information

Transparent Peer Review file
