# Supplementary material for: Rational dsRNA design, scalable production and nanodelivery to enhance spray-induced gene silencing
Source: Commun Biol. 2026 Jul 14;9:953. doi: 10.1038/s42003-026-10663-5 (PMC13369902; doi:10.1038/s42003-026-10663-5)
Supplement: Supplementary file 1 — Transparent Peer Review file [file 42003_2026_10663_MOESM1_ESM.pdf]

nature portfolio

Peer Review File

Rational dsRNA Design, Scalable Production and Nanodelivery to Enhance Spray-Induced Gene Silencing

Corresponding Author: Dr Tao Zhang

This file contains all reviewer reports in order by version, followed by all author rebuttals in order by version.

Version 0:

Reviewer comments:

Reviewer #1

(Remarks to the Author)
I was intrigued to read this review article and found it very inciteful. I enjoyed reading about the origins of RNA technologies and the advance of spray technology in particular. I hope this manuscript is published in Communications Biology. The following comments are meant to improve the quality of the manuscript:

1. Lines 115, 116: you describe the use of HIGS to create transgenic lines that are resistant to pathogens. Please list some references. Are you referring to using spray technology to actually produce transgenic lines? Or are you referring to changing phenotypic traits transiently?

2. lines 347 and 356, use the term "is also meaningful" twice in a row.

3. Line 392, diseases.6?

4. Figure 3, you mention the use of phages as delivery vehicles for mRNA. Are not plant viruses delivery vehicles? Here is a reference for you: https://www.nature.com/articles/s41477-021-00851-y

Some mention of Yuri Gleba's work is in order here, even if it is only a sentence or two.

Reviewer #2

(Remarks to the Author)
Comments:

The manuscript is well written and organized, but few things are needed to be taken care of.

1.The manuscript is mostly descriptive and does not provide enough critical analysis.
Much of the review presents one example after another on HIGS, SIGS, dsRNA design, production methods, and nanocarriers, but it rarely goes beyond summarizing published work. A stronger review should also evaluate the literature more critically by asking which results are broadly reliable, which are limited to specific systems, where studies show inconsistent findings, and which conclusions are still preliminary or difficult to apply under field conditions. For instance, Table 1 mainly lists successful reports, but it does not help the reader compare the quality of evidence, reproducibility, limitations, or practical significance of those studies. Because of this, the manuscript reads more like a collection of literature examples than a truly critical review.

2.The manuscript does not provide the integrated perspective suggested by the title.
The title gives the impression that the review will present a connected framework linking dsRNA design, large-scale production, and nano delivery. However, in the manuscript, these topics are mostly discussed as separate sections rather than as parts of one coordinated strategy. The review does not clearly explain how choices made during dsRNA design may influence production feasibility, how formulation approaches may need to differ depending on the uptake barriers of different target organisms, or how delivery and stability challenges should guide target selection and design decisions. Because of this, the manuscript feels more like a set of related topics placed side by side than a truly integrated and comprehensive

review.

3.The discussion of safety, regulation, and non-target risks is too limited for a topic like this. The manuscript repeatedly presents SIGS as an environmentally friendly and promising tool for agriculture, but the discussion of biosafety is quite short. Non-target effects are considered mainly in terms of sequence similarity, and the safety of nanocarriers is mentioned only in general with little further analysis. Moreover, the review does not adequately address important issues such as regulatory requirements, environmental persistence, residue concerns, exposure risks, effects on non-target organisms, or the practical challenges involved in obtaining approval for agricultural use.

4.The review appears to show a bias toward positive results. Most of the studies discussed in the manuscript highlight successful applications of SIGS, while discrepancy, inconsistent, or low-efficiency outcomes receive little attention. This kind of imbalance could be an issue because it can give an overly optimistic picture of the field. A more balanced discussion should also include studies with limited or variable success, as these are important for understanding the real challenges and current limitations of achieving reliable SIGS performance.

5.The discussion of production cost and platform preference needs clearer support and more careful interpretation. The manuscript makes several statements about the relative cost of different production methods and suggests preference for certain platforms, but these points are not always explained with enough context. Cost estimates can vary depending on availability of technology, production scale, purification steps, and formulation requirements etc. Therefore, such comparisons should be presented more cautiously and supported more clearly, so that readers can better understand the basis and limits of these claims.

Version 1:

Reviewer comments:

Reviewer #1

(Remarks to the Author)
I am satisfied with the revisions made to the manuscript.

Reviewer #2

(Remarks to the Author)
The authors respond to most of my queries that I had asked.
However, one important thing is missing in the current version of the manuscript (MS). The current MS does not adequately address the issue of scalable production of dsRNA, a critical part of the successful application of SIGS in field level. Although they described some methods of dsRNA synthesis. However, a dedicated sub-heading and appropriate description are necessary for scalable production of dsRNA for agricultural use and a comparative discussion of different methods of dsRNA production in view of current knowledge and future ways forward.

Version 2:

Reviewer comments:

Reviewer #2

(Remarks to the Author)
I do not have any further query about the current version of the manuscript.

Open Access This Peer Review File is licensed under a Creative Commons Attribution 4.0 International License, which permits use, sharing, adaptation, distribution and reproduction in any medium or format, as long as you give appropriate credit to the original author(s) and the source, provide a link to the Creative Commons license, and indicate if changes were made.
In cases where reviewers are anonymous, credit should be given to 'Anonymous Referee' and the source.
The images or other third party material in this Peer Review File are included in the article's Creative Commons license, unless indicated otherwise in a credit line to the material. If material is not included in the article's Creative Commons license and your intended use is not permitted by statutory regulation or exceeds the permitted use, you will need to obtain permission directly from the copyright holder.
To view a copy of this license, visit https://creativecommons.org/licenses/by/4.0/

Dear reviewers,

Thank you for providing us with the opportunity to revise our manuscript entitled “Enhancing Spray-Induced Gene Silencing: From Rational dsRNA Design to Scalable Production and Nanodelivery” (Manuscript ID: COMMSBIO-25-11777).

We would like to express our sincere gratitude to the reviewers for their insightful comments and constructive suggestions, which have been immensely helpful in improving the quality and balance of our review. We have carefully addressed all the points raised by the reviewers and have incorporated the necessary revisions into the manuscript. Specifically, we have enhanced the critical analysis of SIGS limitations, refined the cost-benefit discussion of production platforms, and added a new section on biosafety and regulations to meet the journal's high standards.

All changes in the manuscript have been highlighted using yellow background. Our point-by-point responses to the editorial comments are provided below.

To Reviewer#1:

1. Lines 115, 116: you describe the use of HIGS to create transgenic lines that are resistant to pathogens. Please list some references. Are you referring to using spray technology to actually produce transgenic lines? Or are you referring to changing phenotypic traits transiently?

We agree with the reviewer that the original phrasing could lead to a misunderstanding regarding the relationship between spray technology and transgenic lines.

To clarify this, we have reorganized the text in the revised manuscript . We now first define SIGS as a non-transgenic, exogenous application that transiently modifies phenotypic traits. Following this, we introduce HIGS with the phrase "In contrast, HIGS is a transgenic strategy," explicitly stating that it requires the stable integration of DNA constructs into the host genome to produce dsRNA (Lines 93-100).:

SIGS is an exogenous application strategy in which dsRNA or amiRNA, synthesized in vitro or in vivo, is directly sprayed onto crops. A portion of the applied RNA persists on the plant surface, whereas another portion can penetrate plant cells through stomata and be transported throughout various plant parts [15,16]. Although SIGS still faces limitations in terms of RNA stability and cellular uptake efficiency, it eliminates the need for time-consuming transgenic plant breeding, increasing the speed and flexibility of its application.

---

2. lines 347 and 356, use the term "is also meaningful" twice in a row.

We appreciate the reviewer’s attention to detail regarding the repetitive phrasing.

To address this, we have removed the redundant description in Line 367 to ensure the discussion remains concise and focused. These adjustments streamline the narrative and avoid the repetitive use as suggested.

Fungi and insects sometimes also take in miRNAs and siRNAs processed by plants [92]. For example……

---

3. Line 392, diseases.6?

We apologize for this typographical error. The number "6" in Line 421 was an accidental insertion and has been completely removed in the revised manuscript. We have also performed a thorough check of the entire text to ensure no similar formatting artifacts remain.

…… against pests and diseases. Currently, the cost of synthesizing dsRNA ……

---

4. Figure 3, you mention the use of phages as delivery vehicles for mRNA. Are not plant viruses delivery vehicles? Here is a reference for you: https://www.nature.com/articles/s41477-021-00851-y

Some mention of Yuri Gleba's work is in order here, even if it is only a sentence or two.

We thank the reviewer for pointing out the potential of plant viruses as delivery vehicles and for providing this excellent reference (Nature Plants, 2021).

After carefully reviewing the article, we agree that plant viral vectors represent a powerful tool for dsRNA delivery and amplification. However, as the mechanism described in the paper is conceptually more aligned with Virus-Induced Gene Silencing (VIGS) than the exogenous spray application (SIGS) focused on in Figure 3, we have decided to incorporate this valuable content into the Introduction/VIGS section of the revised manuscript (line91-93).:

Furthermore, this technology has been further extended to enable gene expression/overexpression, moving beyond simple gene silencing[14]

To reviewer#2:

1. The manuscript is mostly descriptive and does not provide enough critical analysis.
Much of the review presents one example after another on HIGS, SIGS, dsRNA design, production methods, and nanocarriers, but it rarely goes beyond summarizing published work. A stronger review should also evaluate the literature more critically by asking which results are broadly reliable, which are limited to specific systems, where studies show inconsistent findings, and which conclusions are still preliminary or difficult to apply under field conditions. For instance, Table 1 mainly lists successful reports, but it does not help the reader compare the quality of evidence, reproducibility, limitations, or practical

significance of those studies. Because of this, the manuscript reads more like a collection of literature examples than a truly critical review.

We sincerely thank the reviewer for this insightful and constructive critique. We agree that a high-quality review should move beyond mere summaries to provide a critical evaluation of the field's current state. Accordingly, we have performed a major revision to incorporate more depth and critical analysis throughout the manuscript:

Enhanced Critical Analysis in the Main Text(lines 118-128) :

Despite the robust efficacy observed in controlled environments, the transition of HIGS and SIGS from lab-to-field remains constrained by significant performance gaps. Although substantial gene silencing and phenotypic resistance have been documented, the reproducibility of these results is often hampered by inconsistencies in dsRNA internalization, environmental degradation, and the inherent variability of RNAi machinery across different pathogens. SIGS, in particular, offers the advantage of bypassing transgenic intervention but faces heightened challenges in maintaining dsRNA stability and ensuring effective penetration under heterogeneous field conditions. Such constraints necessitate comprehensive, multi-factorial refinement to establish SIGS as a resilient and potent platform for sustainable agricultural biosecurity.

Revision of Table 1: We have significantly updated Table 1 to include a dedicated column for "Field-scale Evaluation"：

Table 1 Advances in research on RNAi technology in disease control and prevention

Pathogen	Host plant	Target gene	Results	Field test	Methonds	Reference
Diplocarpon coronariae	apple	*Hsp90*	Detached leaf assays showed reduced disease symptoms in transgenic lines compared to wild-type controls.	No	HIGS	(Kumar et al., 2025)
Phytophthora infestans	potato	*ALS*	The downregulation of the ALS gene resulted in significantly increased resistance to Late Blight.	Yes	HIGS	(Temme et al., 2025)
Aphis gossypii	cotton	*AgDPPS1*	The efficacy of the treatment increased cotton aphid mortality rates substantially under both laboratory and field conditions.	Yes	HIGS	(Tian et al., 2025)
Fusarium fujikuroi	rice	*FfCna1, FfCnb1*	The plant exhibited increased resistance to rice Bakanae Disease and displayed a mitigation of disease symptoms.	No	HIGS	(Hou et al., 2025)
Fusarium sacchari	Sugarcane	*FsCYP51*	The transgenic sugarcane lines demonstrated robust resistance against Fusarium infection.	Yes	HIGS	(Yin et al., 2025)
Sporisorium scitamineum	Sugarcane	*SsGlcP*	Transgenic sugarcane lines demonstrated a significant reduction in the biomass of the endophytic basidiomycete.	Yes	HIGS	(Wu et al., 2025)
Verticillium dahliae	cotton	*VdThit*	The transgenic RNAi cotton lines exhibited mitigated disease symptoms accompanied by a significant reduction in Verticillium dahliae biomass.	Yes	HIGS	(Wang et al., 2024)
Rhizoctonia solani AG1-IA	rice	*AGLIP1*	The treatment resulted in the suppression of mycelial growth and biomass, leading to a 57.3% reduction in lesion area on the host plant.	No	HIGS	(Mei et al., 2024)
Fusarium oxysporumf. sp.*Lycopersici*	tomato	*FoFLP1, FoFLP4, FoFLP5*	A decrease in the Disease Index was observed, correlating with a 2.4- to 7.5-fold reduction in FoFLP gene expression within the pathogen.	No	HIGS	(Chauhan and Rajam, 2024)
Fusarium oxysporum f.sp. *Lycopersici*	tomato	*FolRDR1*	The treatment resulted in the attenuation of mycelial growth and a reduction in biomass for *Fusarium oxysporum*, and markedly suppressed the growth of Botrytis cinerea.	No	SIGS	(Ouyang et al., 2023)
Botrytis cinerea	Tobacco, tomato	*BcTRE1*	The significant suppression of Botrytis cinerea growth enhanced the plant's resistance to Gray Mold disease.	No	SIGS	(Jin et al., 2025)
Leaf curl disease-causing begomoviruses	Tobacco	Av1, Av2, Ac1, Ac4	The method led to a significant decrease in viral accumulation within tobacco plants, resulting in the mitigation of the characteristic leaf-curling symptoms.	No	SIGS	(Priyanka et al., 2025)
Magnaporthe oryzae	rice	*MoDES1*	The treatment resulted in the significant suppression of Magnaporthe oryzae growth and a corresponding decrease in the lesion area on rice plants.	No	SIGS	(Sarkar and Roy-Barman, 2021)
Sogatella furcifera	rice	*sfAkt*	The treatment resulted in the significant suppression of female ovarian development, which led to a reduction in egg-laying capacity, correlating with a decrease in target gene expression levels.	No	SIGS	(Guo et al., 2025)
Brassicogethes aeneus	Oilseed rape	*αCOP*	The decrease in target gene expression correlated with a 16% mortality rate observed 15 days after initiation of feeding.	No	SIGS	(Willow et al., 2020)
Sitobion avanae	wheat	*CHS1*	Aphid CHS1 gene expression decreased by 45.32%–50.29%; concurrently, both the total number of aphids and the number of molting aphids were significantly reduced, leading to an increase in wheat grain weight.	No	SIGS	(Zhao et al., 2018)
Phyllotreta	Chinese	*psJHBP-like* ,	Reduced gene expression resulted in a	No	SIGS	(Xu et al.,

Pathogen	Host plant	Target gene	Results	Field test	Methonds	Reference
striolata	cabbage	*psaaNAT-like*	decrease in feeding rate			2025)
Verticillium dahliae	cotton	*PMT*	The treatment inhibited the growth of Verticillium dahliae, leading to alleviated symptoms of cotton Verticillium Wilt.	No	MIGS	(Wen et al., 2023)

Restructured Outlook: The Outlook section has been completely rewritten to provide a more critical roadmap for the field. We now focus on the bottleneck problems rather than just future possibilities (lines 734-762)：

The primary imperative is the continuous optimization of dsRNA design and the enhancement of target prediction accuracy. Contrary to earlier assumptions, emerging evidence suggests that dsRNA length is not the definitive determinant of resistance. Instead, uptake efficiency and environmental context often overshadow intrinsic design parameters. These data must ultimately be integrated into efficient, automated design platforms to develop more versatile and potent dsRNA molecules.

The transition from laboratory-scale yields to industrial-scale production remains a significant hurdle. Many laboratory-proven yields have yet to translate reliably to commercial manufacturing. Although engineered microbial and cell-free systems have reduced costs, scaling is frequently hampered by plasmid instability and inducer toxicity during fermentation. Achieving consistent, low-energy, and standardized processes capable of meeting the agricultural demand for dsRNA requires further optimization.

Most critically, the development of efficient and safe nanodelivery systems remains a primary bottleneck. The performance of nanocarriers under field conditions often falls short of results observed in controlled laboratory trials. Formulations that appear robust in vitro tend to degrade rapidly when exposed to environmental stressors such as UV radiation, precipitation, and temperature fluctuations. Furthermore, comprehensive environmental and human safety assessments are still lacking for many formulations; the biosafety risks associated with long-term soil accumulation, potential impacts on non-target organisms (e.g., beneficial microbiota and pollinators), and unintended off-target gene silencing remain largely unknown. Addressing these obstacles through multi-location field trials and advanced nanocarriers is essential for transitioning RNAi technology from proof-of-concept studies to large-scale agricultural adoption.

Ultimately, the synergy between multi-factorial dsRNA design, scalable synthesis, and precision delivery will define the future commercial viability of SIGS-based solutions.

2. The manuscript does not provide the integrated perspective suggested by the title.

The title gives the impression that the review will present a connected framework linking dsRNA design, large-scale production, and nano delivery. However, in the manuscript, these topics are mostly discussed as separate sections rather than as parts of one coordinated strategy. The review does not clearly explain how choices made during dsRNA design may influence production feasibility, how formulation approaches may need to differ depending on the uptake barriers of different target organisms, or how delivery and stability challenges should guide target selection and design decisions. Because of this, the manuscript feels more like a set of related topics placed side by side than a truly integrated and comprehensive review.

We are grateful for the reviewer's perspective on the need for a more integrated framework. We agree that the true value of a "design-to-delivery" review lies in the interplay between these stages. To address this, we have incorporated several new discussion sections to bridge the gaps between previously separate topics:

Connecting Design with Production (Lines 388–397), (Lines 652–661): We have added a discussion on how molecular design choices, such as the length of the dsRNA or the inclusion of specific secondary structures, directly impact the metabolic load and yield during microbial fermentation or the efficiency of cell-free synthesis:

In summary, while multiple dimensions have been identified for designing efficient and stable dsRNA for SIGS. However, they must be harmonized with downstream production and delivery constraints to be truly viable. For instance, although longer dsRNAs (>500 nt) may theoretically enhance silencing potency, they often compromise bacterial expression yields—due to transcriptional collisions or plasmid instability—and complicate uniform loading into nanocarriers. Currently, the empirical data bridging specific dsRNA structural traits with large-scale manufacturing and nano-delivery remains insufficient. Consequently, establishing a holistic design framework that accounts for these downstream bottlenecks remains a significant challenge for the field.

The selection of an optimal nanocarrier is a sophistic amentally governed by the structural morphology and electrochemical profile of the dsRNA. Specifically, the length of the dsRNA and its charge distribution play decisive roles in determining the success of the delivery system. Shorter dsRNA molecules, particularly those engineered to target highly accessible regions, demonstrate significantly higher loading efficiency and more robust intercalation within LDH nanosheets[142]. Furthermore, to ensure high payload capacity and prevent competitive binding, rigorous downstream purification is often essential before the complexation process can proceed, ensuring the resulting nano-assemblies are both stable and biologically potent [143].

3. The discussion of safety, regulation, and non-target risks is too limited for a topic like this. The manuscript repeatedly presents SIGS as an environmentally friendly and promising tool for agriculture, but the discussion of biosafety is quite short. Non-target effects are considered mainly in terms of sequence similarity, and the safety of nanocarriers is mentioned only in general with little further analysis. Moreover, the review does not adequately address important issues such as regulatory requirements, environmental persistence, residue concerns, exposure risks, effects on non-target organisms, or the practical challenges involved in obtaining approval for agricultural use.

We agree with the reviewer that a comprehensive discussion on biosafety and regulatory frameworks is indispensable for a review on SIGS technology. We appreciate this suggestion to broaden the scope and depth of our analysis in these critical areas.

To address these concerns, we have added a major new section (lines 670-729).:

Biosafety, Regulatory Considerations, and Environmental Risk Assessment

Although spray-induced gene silencing (SIGS) is recognized as a highly specific and eco-friendly alternative to conventional chemical pesticides, its successful commercialization hinges upon rigorous biosafety evaluations. While the sequence-specific nature of RNAi provides an inherent safety margin, establishing a sustainable deployment framework requires a meticulous assessment of potential off-target effects, the impact of nanocarriers on environmental persistence, and human exposure risks.

The specificity of SIGS stems from sequence complementarity, where well-curated dsRNAs minimize homology to non-target genomes through stringent bioinformatics filtering. However, off-target silencing remains a possibility if siRNAs share sufficient matches with transcripts in phylogenetically related species. Exposure pathways for non-target organisms (NTOs) include direct contact, trophic transfer, and indirect movement through soil or aquatic systems. While closely related taxa are most susceptible, distant lineages often exhibit reduced sensitivity due to divergent RNAi machinery or physiological uptake barriers. Consequently, a tiered risk assessment is imperative[147]. Furthermore, while multi-target designs enhance efficacy, they necessitate broader off-target screening. Overall, current evidence suggests that risks are minimal when sequences are judiciously selected, though case-by-case evaluation remains a fundamental requirement.

Nanomaterials significantly enhance dsRNA delivery and protection but introduce additional ecological considerations. Carriers such as chitosan, SPc, and LDH generally exhibit favorable biodegradability and low acute toxicity at field-relevant dosages. Nevertheless, they may extend the environmental persistence of dsRNA, facilitate unintended uptake in NTOs, or release specific carrier components[148]. Therefore, ecotoxicological testing—encompassing chronic effects on beneficial organisms and life-cycle assessments—must be integrated into the

development process. Despite existing data gaps regarding long-term field behavior, current risks appear manageable at proposed application rates.

Human exposure is primarily restricted to occupational routes during spray application, with negligible dietary risk. Mammals possess robust biological barriers to dsRNA activity, including salivary and gastrointestinal nucleases and highly acidic gastric environments. Currently approved products are classified as practically non-toxic (EPA Toxicity Category IV). While nanocarriers may subtly alter bioavailability, necessitating specific gastric stability data[149]. Therefor, appropriate personal protective equipment and low-drift application techniques will further minimize risks.

In the United States, the EPA regulates externally applied dsRNA products as biochemical pesticides under FIFRA and FFDCA. Assessments follow a tiered, case-by-case approach focusing on bioinformatics, environmental fate, and limited NTO testing. A landmark approval is Calantha™ , the first sprayable dsRNA insecticide registered in 2023–2024 for control of Colorado potato beetle on potatoes[150]. It received a conditional registration supported by data showing rapid degradation, negligible residues, and low risk to human health and the environment.

The European Union often applies stricter scrutiny, sometimes under novel biotechnology frameworks requiring extended ecological data. OECD guidance documents on environmental and human health risk assessment for externally applied dsRNA pesticides provide harmonized frameworks for data requirements and exposure pathways[149,151]. Key commercialization challenges include the lack of fully standardized protocols for nano-formulated products, high assessment costs, lab-to-field consistency, public perception of “genetic sprays,” and resistance management. Transparent, science-based evaluations are essential for building regulatory confidence and global acceptance.

In summary, SIGS offers significant specificity and reduced environmental load compared with broad-spectrum chemicals. Its responsible adoption requires continued rigorous, transparent biosafety research and adaptive regulatory oversight. Through interdisciplinary collaboration among molecular biologists, toxicologists, agronomists, and regulators, RNAi technology can realize its potential as a truly sustainable tool for crop protection.

---

4. The review appears to show a bias toward positive results. Most of the studies discussed in the manuscript highlight successful applications of SIGS, while discrepancy, inconsistent, or low-efficiency outcomes receive little attention. This kind of imbalance could be an issue because it can give an overly optimistic picture of the field. A more balanced discussion should also include studies with limited or variable success, as these are important for

understanding the real challenges and current limitations of achieving reliable SIGS performance.

We agree that a balanced review must address not only the successes but also the inconsistencies and inherent limitations of SIGS technology to provide a realistic roadmap for the field.

In the revised manuscript, we have systematically integrated discussions of low-efficiency, inconsistent, and species-specific outcomes to provide a more objective perspective:

Addressing Target Selection and Stability (Lines 168–174):

While the majority of SIGS studies demonstrate that targeting a single essential gene can effectively confer pathogen resistance, the degree of disease control often remains highly dependent on the specific target selected. For instance, in the management of Phytophthora infestans, although spraying dsRNA targeting genes like *PiGPB1*, *PiHmp1*, or *PiEndo3* successfully mitigated late blight symptoms, the resulting levels of suppression were markedly inconsistent across different genetic targets[45].

Biological Barriers and Uptake Efficiency (Lines 204–209):

However, the feasibility of such broad-spectrum strategies is fundamentally constrained by the inherent variability in RNA uptake efficiencies across species. For instance, while pathogens like Botrytis cinerea and Verticillium dahliae exhibit high dsRNA uptake, others such as Colletotrichum gloeosporioides show no uptake at all, and even the oomycete Phytophthora infestans displays limited and cell-type-specific absorption[53].

Addressing Environmental and Material Variability (Lines 666–669):

Nevertheless, the performance of various nanomaterials is highly contingent upon the specific species and application environment. This inherent variability necessitates the screening of multiple nanomaterial candidates to guarantee reliable SIGS-mediated protection[144,145]

---

5. The discussion of production cost and platform preference needs clearer support and more careful interpretation. The manuscript makes several statements about the relative cost of different production methods and suggests preference for certain platforms, but these points are not always explained with enough context. Cost estimates can vary depending on availability of technology, production scale, purification steps, and formulation requirements etc. Therefore, such comparisons should be presented more cautiously and supported more clearly, so that readers can better understand the basis and limits of these claims.

We agree that cost estimates are highly sensitive to multiple variables and that a more cautious, context-aware interpretation is necessary.

In the revised manuscript (Lines 431–445), we have refined our discussion on production platforms to provide a more balanced and transparent analysis:

However, these cost estimates should be interpreted with caution. Reported figures often reflect optimized pilot- or industrial-scale processes and may not fully account for additional expenses associated with downstream purification, formulation with nanocarriers, quality control, or regulatory compliance. Actual costs can vary significantly depending on production scale, geographic location, availability of infrastructure, energy inputs, and the specific dsRNA sequence. Moreover, challenges such as plasmid instability, inducer toxicity, and batch-to-batch variability in microbial systems still require further optimization for consistent industrial performance.

Nevertheless,considering the practical demands of large-scale production, microbial in vivo synthesis or the use of cell-free platforms built with endogenous cellular components are the preferred options for the economic and high-yield synthesis of dsRNA. Therefore, the implementation of SIGS must prioritize optimizing the use of engineered microorganisms, protective carriers, and the overall protection of dsRNA.

We hope that the extensive revisions we have made successfully address the concerns raised and that the manuscript is now suitable for publication. We look forward to hearing from you at your earliest convenience.

Sincerely,

Tao Zhang, Ph.D.
Corresponding Author
Xinjiang Institute of Ecology and Geography, Chinese Academy of Sciences
Email: tzhang@ms.xjb.ac.cn

Dear Reviewers,

Thank you for your careful reading of our manuscript and for your valuable comments. We have carefully considered all the points raised and have revised the manuscript accordingly. In response to Reviewer #2's suggestion, we have added a new independent section dedicated to the scalable production of dsRNA, including a comparative discussion of different production methods and future perspectives. All changes have been highlighted in the revised manuscript. Below are our point-by-point responses to each reviewer's comments.

Reviewer #1

I am satisfied with the revisions made to the manuscript.

Response: We greatly appreciate Reviewer's positive evaluation of our revisions.

Reviewer #2

The authors respond to most of my queries that I had asked.
However, one important thing is missing in the current version of the manuscript (MS). The current MS does not adequately address the issue of scalable production of dsRNA, a critical part of the successful application of SIGS in field level. Although they described some methods of dsRNA synthesis. However, a dedicated sub-heading and appropriate description are necessary for scalable production of dsRNA for agricultural use and a comparative discussion of different methods of dsRNA production in view of current knowledge and future ways forward.

Response: We thank Reviewer for raising this important point. We fully agree that scalable production is a key bottleneck for the agricultural application of SIGS. To address this, we have added a new independent section in the revised manuscript entitled "Production of dsRNA: Current Methods, Comparison, and Future Directions". We believe this new section fully addresses the reviewer's concern regarding scalable production and comparative discussion. All new text has been highlighted in the manuscript.

The corresponding modifications can be found on lines 416–467 of the revised manuscript.

Production of dsRNA: Current Methods, Comparison, and Future Directions

Although the methods described above provide a solid technical foundation for dsRNA production, their practical application in agriculture remains limited. It is estimated that 2 to 10 grams of dsRNA per hectare are required for effective field spray control of major agricultural pests and diseases. Consequently, the key to successful commercialization of SIGS lies in the ability to produce high-quality dsRNA at low cost and on a large scale.

Current production methods vary significantly in cost and scalability. Chemical synthesis, while highly precise, is prohibitively expensive for long dsRNA sequences. Conventional IVT is widely used in laboratory settings; however, the high cost of nucleotide substrates, enzymes, and purification equipment results in pilot-scale production costs of approximately $50–$60 per gram, making large-scale field application economically unfeasible[100]. Microbial fermentation using engineered microorganisms has emerged as a more cost-effective alternative. Systems based on RNase III-deficient *E. coli* or *Corynebacterium glutamicum* can achieve yields of 0.3–1.0 g/L, with pilot-scale costs potentially dropping to $2–$5 per gram [101].. These platforms benefit from mature fermentation infrastructure and relatively low raw material costs. Nevertheless, challenges such as plasmid instability, batch-to-batch variability, and stringent downstream purification requirements remain.

Cell-free synthesis platforms have demonstrated strong potential for agricultural scale-up. GreenLight Biosciences has successfully commercialized its proprietary cell-free system, which utilizes cell extracts, enzymes, and short DNA templates to produce Calantha™ (Ledprona)—the first sprayable dsRNA bio-insecticide approved by the U.S. Environmental Protection Agency (EPA)[149]. This platform reportedly achieves production costs approaching or below $0.5–$1 per gram at commercial scale, while offering high sequence flexibility and minimal infrastructure requirements.[102]..

It is important to interpret these cost estimates with caution. The reported figures are typically based on optimized pilot or industrial processes and may not fully account for additional expenses such as downstream purification, nano-carrier formulation, quality control, and regulatory compliance. Actual costs can vary substantially depending on production scale, geographic location, infrastructure availability, energy prices, and the specific dsRNA sequence. Furthermore, issues including plasmid instability, inducer toxicity, and batch-to-batch variability in microbial systems still require further optimization to ensure consistent industrial production.

In summary, no single production platform is perfect. However, it is clear that microbial fermentation and cell-free synthesis will be the dominant systems for future agricultural applications of SIGS, replacing chemical synthesis. At present, microbial fermentation offers the most practical route for near-term scale-up, while cell-free systems hold greater promise for long-term cost reduction and regulatory acceptance. Hybrid approaches that combine microbial enzyme production with cell-free assembly are likely to emerge as the optimal solution.

In practice, platform selection should be guided by comprehensive techno-economic analyses that consider not only raw production costs but also the total cost within real-world agricultural supply chains. Therefore, advancing SIGS requires priority focus on optimizing engineered microorganisms, expression vectors, and dsRNA protection strategies to enable lower-cost and more efficient field applications.

Once again, we thank the reviewer for this constructive suggestion, which has significantly improved the completeness and practical value of our manuscript.

We thank all reviewers again for their time and professional guidance.

Sincerely,

Tao Zhang
Corresponding author
Xinjiang Institute of Ecology and Geography, Chinese Academy of Sciences
tzhang@ms.xjb.ac.cn
